# Impact of travel ban implementation on COVID-19 spread in Singapore, Taiwan, Hong Kong and South Korea during the early phase of the pandemic: a comparative study

**DOI:** 10.1186/s12879-021-06449-1

**Published:** 2021-08-11

**Authors:** Sylvia Xiao Wei Gwee, Pearleen Ee Yong Chua, Min Xian Wang, Junxiong Pang

**Affiliations:** 1 Saw Swee Hock School of Public Health, National University of Singapore and National University Health System, Singapore, 117549 Singapore; 2 Centre for Infectious Disease Epidemiology and Research, National University of Singapore, Singapore, 117549 Singapore

**Keywords:** COVID-19, Imported case, Asia, Transmission

## Abstract

**Background:**

The COVID-19 pandemic has elicited imposition of some form of travel restrictions by almost all countries in the world. Most restrictions currently persist, although some have been gradually eased. It remains unclear if the trade-off from the unprecedented disruption to air travel was well worth for pandemic containment.

**Method:**

A comparative analysis was conducted on Singapore, Taiwan, Hong Kong and South Korea’s COVID-19 response. Data on COVID-19 cases, travel-related and community interventions, socio-economic profile were consolidated. Trends on imported and local cases were analyzed using computations of moving averages, rate of change, particularly in response to distinct waves of travel-related interventions due to the outbreak in China, South Korea, Iran & Italy, and Europe.

**Results:**

South Korea’s travel restrictions were observed to be consistently more lagged in terms of timeliness and magnitude, with their first wave of travel restrictions on flights departing from China implemented 34 days after the outbreak in Wuhan, compared to 22–26 days taken by Singapore, Taiwan and Hong Kong. South Korea’s restrictions against all countries came after 91 days, compared to 78–80 days for the other three countries. The rate of change of imported cases fell by 1.08–1.43 across all four countries following the first wave of travel restrictions on departures from China, and by 0.22–0.52 in all countries except South Korea in the fifth wave against all international travellers. Delayed rate of change of local cases resulting from travel restrictions imposed by the four countries with intrinsic importation risk, were not observed.

**Conclusions:**

Travel restriction was effective in preventing COVID-19 case importation in early outbreak phase, but may still be limited in preventing general local transmission. The impact of travel restrictions, regardless of promptness, in containing epidemics likely also depends on the effectiveness of local surveillance and non-pharmaceutical interventions concurrently implemented.

**Supplementary Information:**

The online version contains supplementary material available at 10.1186/s12879-021-06449-1.

## Background

Since the beginning of the COVID-19 pandemic, WHO has consistently denounced restrictions of international traffic, and only recommends measures to limit the risk of exportation and importation [1]. Travel bans were introduced in the past to control the spread of the viruses, such as those on Mexico during the 2009 H1N1 pandemic [2, 3], on West Africa during the 2014 Ebola [4] and on influenza [5]. Nonetheless, countries descended into a flurry imposing travel restrictions of varying scales. Travel restrictions varies across a spectrum, generally classified as border closure, entry or exit ban, visa suspension, transport suspension, arrival quarantine, border screening and other special entry procedure in decreasing magnitude of restrictiveness.

The limitations of travel bans are often only well elucidated through mathematical modelling papers [5]. Quantitative evaluation of their effectiveness is hindered by the inconsistent implementation of other non-pharmaceutical measures adapted to suit different nations. Nonetheless, studies over the years were congruent in proving that almost a 100% shutdown of global travel is required to achieve only modest to negligible effect in delaying the arrival of imported cases or pandemic spread in most cities [3, 6–9]. In addition, the implementation of travel bans to limit importation risk may seem intuitive theoretically, but is a highly disruptive, costly and resource-intensive measure to implement in reality [10–12]. It could also lead to dire economic consequences for the affected countries, particularly less developed nations, dis-incentivizing them from reporting future outbreaks. A few travelling infected individuals are sufficient to seed epidemics in other countries, triggering global spread even as the epidemic is mitigated and appears under control at its source [13]. Thus, travel restrictions are almost ineffective in slowing the pandemic evolution if epidemics are not controlled at its source. Robust local control is more likely able to reduce virus transmission [3, 6], as travel restrictions are often implemented belatedly.

However, countries have resorted to travel restrictions, possibly under political pressure more so than for public health benefits. This is especially plausible in the absence of pharmaceutical circumventions and mounting public pressure against the imminent threat of a novel virus [14, 15]. As of 30th June 2020, the Centre of Infectious Disease Epidemiology & Research in Saw Swee Hock School of Public Health identified a total of 104 WHO/non-WHO states and territories imposing travel restriction on China alone, excluding the numerous restrictions against other heavily affected areas [16]. In May 2020, Singapore’s Changi Airport reportedly handled approximately 100 arrivals and 700 departures a day [17], compared to more than 170,000 passengers daily in May 2019. Daily flights have also dwindled from 7400 before the outbreak to just 80.

The close geographical proximity, business travel, tourism and supply link of Southeast Asia with China increase the risk of case importation [18]. This contributed to Southeast Asia being one of the first regions affected with COVID-19. However, there is limited study on the impact of travel ban as an early defense against COVID-19 among Asian countries [19]. Hence, this study aims to assess the impact of travel bans imposed by four Asian countries, namely Singapore, Taiwan, Hong Kong and South Korea against COVID-19 through a comparative analysis. In particular, the study focuses on travel restrictions targeted at the European countries as Europe emerged as the new pandemic epicenter in mid-March 2020.

## Methods

### Search strategy

Countries in the WHO Western Pacific region [20] were searched to identify data suitable for analysis. For countries providing regular, individualized case update, a further search on their profile and interventions was conducted on their respective government websites. A supplementary Google search was also performed, including findings up to April 5, 2020.

### Data collection

Data on COVID-19 cases were collected from daily press releases by the 4 countries’ respective disease control agencies – Singapore Ministry of Health [21], Taiwan Centers for Disease Control [22], Hong Kong Centre for Health Protection [23], and South Korea Centers for Disease Control and Prevention [24]. Case data were compiled up to April 5, 2020. Travel histories were classified according to UN geoscheme (Africa, Americas, Asia, Europe & Oceania) to match the classification used by the South Korea CDC. Travel related restrictions were extracted from a daily surveillance report monitoring press releases of agencies, press sites of governments, and online news sites [16].

Socioeconomic profiles were evaluated to further understand their pandemic readiness. Countries’ profiles were screened through their respective government websites – Department of Statistic Singapore [25], National Statistics of Republic of China (Taiwan) [26], Census and Statistics Department, Hong Kong Special Administrative Region [27] and Statistics Korea [28]. Updated profile on Korea was obtained from news sites due to the absence of updated 2019 population census [29–31]. Data on air traffic [32–35], doctor per 1000 population ratio [36–39], medical coverage [40–42], Human Development Index [43, 44], Gross Domestic Product [45], Global Health Security Index [46], Joint External Evaluation [47, 48] scores, prior pandemic experience with Severe Acute Respiratory Syndrome, pandemic Influenza A (H1N1–2009) and Middle East Respiratory Syndrome were also reviewed [49–56].

### Analysis

Epidemic curves of disease progression in the four countries were charted on a timeline of travel ban and quarantine on inbound travellers. Their effectiveness were analyzed in five waves, against Wuhan (Wave 1), South Korea (Wave 2), Italy/Iran (Wave 3), Europe (Wave 4) and all countries (Wave 5) respectively. The term Europe-related travel restriction refers to interventions targeting European countries other than Italy.

Given the sporadic counts of imported and local cases in the early stages of the epidemic, considerations on the median incubation period of cases [57] and potential reporting delays, we computed daily cases in terms of 3-, 5-, 7- and 14-day moving averages, with 7-day moving average utilized for our main discussion. Rate of change in imported and local cases were computed using moving averages of daily cases. The calculation for South Korea in mid-March was hindered due to missing data. Pearson’s correlation analysis was conducted on rate of change of imported and local cases in the 7 and 14 days post each wave of intervention. All calculations and graphical presentations were constructed using Excel 2016 and Tableau respectively.

## Results

### Socio-economical-pandemic readiness status

The socioeconomic profiles of the four Asian countries were largely similar. All had very high levels of human development according to the human development index in 2018. Their economic development was comparable, with Singapore ranked first, and followed by Hong Kong, South Korea and Taiwan. Population density ranged widely, with Singapore and Hong Kong being approximately 10 times more populated than Taiwan and South Korea. Elderly population accounted for 10.2 to 17.5% of the total population – the highest was observed in Hong Kong (17.5%), South Korea (15.5%) and Taiwan (14.8%) had similar proportions, and Singapore had the lowest proportion (10.2%). In terms of air traffic, South Korea ranked first with 10,173,294 passengers in March 2019, followed by Hong Kong (6,396,906), Singapore (5,630,780) and Taiwan (4,709,041). Figures for March were referenced to compare with the pandemic peak in 2020 when international travel restrictions mounted. Using doctor per 1000 population ratio as an indicator of healthcare system capacity, Singapore was the most adequately staffed (2.5), followed by Korea (2.2), Hong Kong (1.9) and Taiwan (1.7). Universal health coverage is almost present in all four countries. Singapore employs a multipayer, mixed financing system for its primary public health insurance. Taiwan’s primary public insurance is a single-payer system administered by the government. South Korea’s National Health Insurance System covers 97.1% of the population as of 2016 and Hong Kong’s public healthcare offers comprehensive services to the population at a very low cost, largely funded by government tax and non-tax revenues. Notably, all four countries were significantly impacted during epidemics of SARS-CoV (2002–2003), Influenza A (H1N1–2009) (2009–2010) or MERS-CoV (2015) in the past two decades (Table 1).

**Table 1:** Comparison of interventions taken by country

Country	Singapore		Taiwan		Hong Kong		South Korea	
Population, mid-2019 (persons)	5,703,600		23,591,031		7,507,400		51,830,000	
Human Development Index, 2018	0.935 (9th)		0.911 (21st)^1^		0.939 (4th)		0.906 (22nd)	
Population Density, 2019 (persons per sq. km)	7866		651.7		6940		510.5	
Elderly Proportion, 2019 (%)	10.2		14.9		17.5		15.5	
GDP/Capita, 2018 (USD)	64,581.90		25,893		48,675.60		31,380.10	
Doctor per 1000 population (2019)	2.5		1.7		1.9		2.2 (2017)	
Pandemic Experience (Virus/Case/Mortality)^&^	SARS-CoV^2^ (2002–2003) Cases: 238 Deaths: 33 3rd hardest hit outside China Pandemic influenza A (H1N1–2009) (2009–2010) Cases: ~ 415,000 Deaths: ≥18		SARS-CoV (2002–2003) Cases: 664 Deaths: 73 2nd hardest hit outside China Pandemic influenza A (H1N1–2009) (2009–2010) Cases: 954 Deaths: 46		SARS-CoV (2002–2003) Cases: 1755 Deaths: 298 Hardest hit outside China Pandemic influenza A (H1N1–2009) (2009–2010) Cases: 36,534 Deaths: 80		SARS-CoV Cases: 3 Pandemic influenza A (H1N1–2009) (2009–2010) Cases: 750,000 Deaths: 252 MERS-CoV^3^ (2015) Cases: 186 Deaths: 36 Hardest hit outside Saudi Arabia	
Global Health Security Index^#^	58.7		NA		NA		70.2	
Joint External Evaluation^%^ (Total Score: 240)	221		212		NA		217	
**COVID-19**								
Date of 1st imported case reported in examined country (No. of days since outbreak in Wuhan)	23-Jan-20	22	21-Jan-20	20	23-Jan-20	22	20-Jan-20	19
Date of 1st local case reported in examined country (No. of days since outbreak in Wuhan)	03-Feb-20	33	28-Jan-20	27	30-Jan-20	29	30-Jan-20	29
No. of days between 1st imported case and 1st restriction implemented	0		4		4		15	
Details for first travel-related restriction against following epicenters:								
Epicenters:	Description of travel restriction:							
China (Wave 1)	Cessation of all inbound flights from Wuhan		1. Banned entry of Hubei residents, entry of chinese nationals to Kinmen, Matsu & Penghu Islands 2. Suspension of entry permits for Chinese nationals		Banned entry of Hubei residents		Banned entry of travellers who have been to Hubei within 14 days of arrival	
South Korea (Wave 2)	1. Banned entry/transit of travellers coming from Daegu & Cheongdo 2. 14-day quarantine for inbound residents & long-term pass holders from these two areas		14-day quarantine for foreign travellers coming from South Korea		Banned entry of foreign travellers coming from South Korea		NA	
Iran/Italy (Wave 3)	1. Banned entry of Iranian passport holders 2. Suspension of visas issued to Iranian passport holders^3^		14-day quarantine for travellers coming from Italy^4^		14-day quarantine for travellers coming from Iran, Emilia-Romagna, Lombardy & Veneto in northern Italy		NA^$^	
Europe (Wave 4)	Banned entry of travellers who have visited Italy^4^, France, Spain & Germany in the last 14 days		14-day quarantine for travellers coming from 27 countries of Europe’s Schengen border free travel zone, Britain, Ireland & Dubai (transits included)		14-day quarantine for travellers coming from Italy 2, parts of France, Spain & Germany & Japan		14-day quarantine for long-term stay foreign travellers coming from Europe	
All countries (Wave 5)	14-day quarantine for inbound travellers		1. Banned entry of most travellers, except resident permit holders & diplomats 2. 14-day quarantine for inbound travellers		14-day quarantine for all travellers except those from mainland China, Taiwan & Macau		14-day quarantine for inbound travellers	
Epicenters:	Date of implementation (No. of days first travel restrictions were implemented against following epicenter, since outbreak initiation in a. the respective epicentre^5^ and b. Wuhan^5^):							
China	23-Jan-20	a. 22 b. 22	25-Jan-20	a. 24 b. 24	27-Jan-20	a. 26 b. 26	04-Feb-20	a. 34 b. 34
South Korea	26-Feb-20	a. 2 b. 56	25-Feb-20	a. 1 b. 55	25-Feb-20	a. 1 b. 55	NA	NA
Iran/Italy	03-Mar-20	a. 12/8 b. 62/75	28-Feb-20	a. 8/4 b. 58/73	29-Feb-20	a. 9/5 b. 59/73	NA	NA
Europe	16-Mar-20	a. 2 b.75	14-Mar-20	a. 0 b. 73	14-Mar-20	a. 0 b. 73	22-Mar-20	a. 8 b.81
All countries	21-Mar-20	b. 80	18-Mar-20	b. 77	19-Mar-20	b. 78	01-Apr-20	b. 91
Epicenters:	Between the first travel restriction imposed for the following epicenter and the first travel restriction for the next epicenter i.e. from start of previous wave till next wave: No. of cases reported^6^; Time period (days)							
Outbreak initiation in Wuhan till first travel-related intervention by examined country (Before Wave 1 till Wave 1)	Imported: 0 Local: 0	22	Imported: 3 Local: 0	24	Imported: 8 Local: 0	26	Imported: 8 Local: 7	34
China – South Korea (Between Waves 1 and 2)	Imported: 24 Local: 67	34	Imported: 13 Local: 14	31	Imported: 10 Local: 63	29	NA	NA
South Korea – Iran/Italy (Between Waves 2 and 3)	Imported: 0 Local: 18	6	Imported: 0 Local: 2	3	Imported: 3 Local: 10	4	NA	NA
Iran/Italy – Europe (Between Waves 3 and 4)	Imported: 56 Local: 67	13	Imported: 7 Local: 11	15	Imported: 24 Local: 20	14	NA	NA
Europe – All countries (Between Waves 4 and 5)	Imported: 128 Local: 49	5	Imported: 27 Local: 0	4	Imported: 35 Local: 20	5	Imported: 418 Local: 569	10
All countries – 5 April 2020 (After Wave 5 till cut-off)	Imported: 344 Local: 556	15	Imported: 262 Local: 24	18	Imported: 426 Local: 272	17	Imported: 223 Local: 228	4
Total cases reported as of 5 April 2020 (%)	Imported: 552 (42.2) Local: 757 (57.8)		Imported: 312 (86.0) Local: 51 (14.0)		Imported: 506 (56.8) Local: 385 (43.2)		Imported: 741 (7.2) Local: 9496 (92.8)	

^1^As Taiwan is not a member of the United Nations, the government of Taiwan calculates its own HDI

^2^Severe Acute Respiratory Syndrome Soronavirus, SARS-CoV; Middle Eastern Respiratory Syndrome Coronavirus, MERS-CoV

^3^Singapore followed up with a travel ban for incoming visitors with a travel history to Iran, northern Italy and the Republic of Korea in the past 14 days, on March 5, 2020

^4^Travel restriction applies to inbound arrivals from the entire Italy, not just northern Italy

^5^Outbreak initiation dates for the following epicentres: China (Wuhan), 31-Dec-2019; South Korea, 23-Feb-2020; Iran, 19-Feb-2020; Italy, 23-Feb-2020; Europe (excluding Italy), 13-Mar-2020; outbreak initiation date may refer to the date where the start of increased cases was reported/alert level was raised/epicenter was reported

^6^Cases recorded during each phase were tabulated for mere comparison of epidemic progression in each country, and should not be attributed to the restriction implemented at the start of the phase as cases mostly present themselves after an incubation period of up to 14 days

^&^ Pandemic Influenza A (H1N1–2009) figures accurate as of the following date for each country: Singapore (Unknown), Taiwan (31 July 2010), Hong Kong (19 May 2010), South Korea (August 2010)

^#^ As of 2019

^%^ Latest available information used; Singapore (2018), Taiwan (2016), South Korea (2017)

^$^ While we did not find any travel ban or restriction implemented by South Korea against Italy/Iran, the government did raise the travel alert for 5 Italian provinces to “yellow” warning

*Table interpretation: countries studied are listed in columns while countries/regions listed in rows are the epicentres for which travel bans were implemented against. The first section of the table compares the socio-economic profile and pandemic experience of the countries. The second section on COVID-19 can be interpreted in following 3 sub-sections:

1) Description of the first travel restrictions implemented by the four countries of interest (columns) against the epicentres (rows);

2) Date in which the first travel restrictions (described in 1) were implemented by the four countries of interest (columns) against the epicentres (rows), and the corresponding number of days it occurred since outbreak initiated in the epicentre and Wuhan respectively;

3) Number of imported/local cases reported during each wave (defined as the period between implementation of first travel restriction against one epicentre and the next epicentre) and the number of days in each wave

### Epidemic trend and progression

All four countries had distinct disease progression since the report of their first case. South Korea reported the highest number of cases (10,237), with only 7.2% imported. Singapore confirmed 1309 cases, 42.2% of which were imported; Hong Kong confirmed 891 cases, 56.8% were imported; and Taiwan confirmed 363 cases, 86% were imported. (Table 1). All countries confirmed their first imported infections from Wuhan, China between January 20 and 23, 2020. Considering differences in population sizes, incidence of imported cases in the countries (per 100,000 population) were 9.68, 1.32, 6.74 and 1.43 for Singapore, Taiwan, Hong Kong and South Korea respectively. Despite South Korea recording the greatest absolute number of imported cases as of April 5, Singapore had the highest imported incidence rate due to its small population size (Fig. 6).

### Country specific case trend and travel restriction progression

**Fig. 1:**
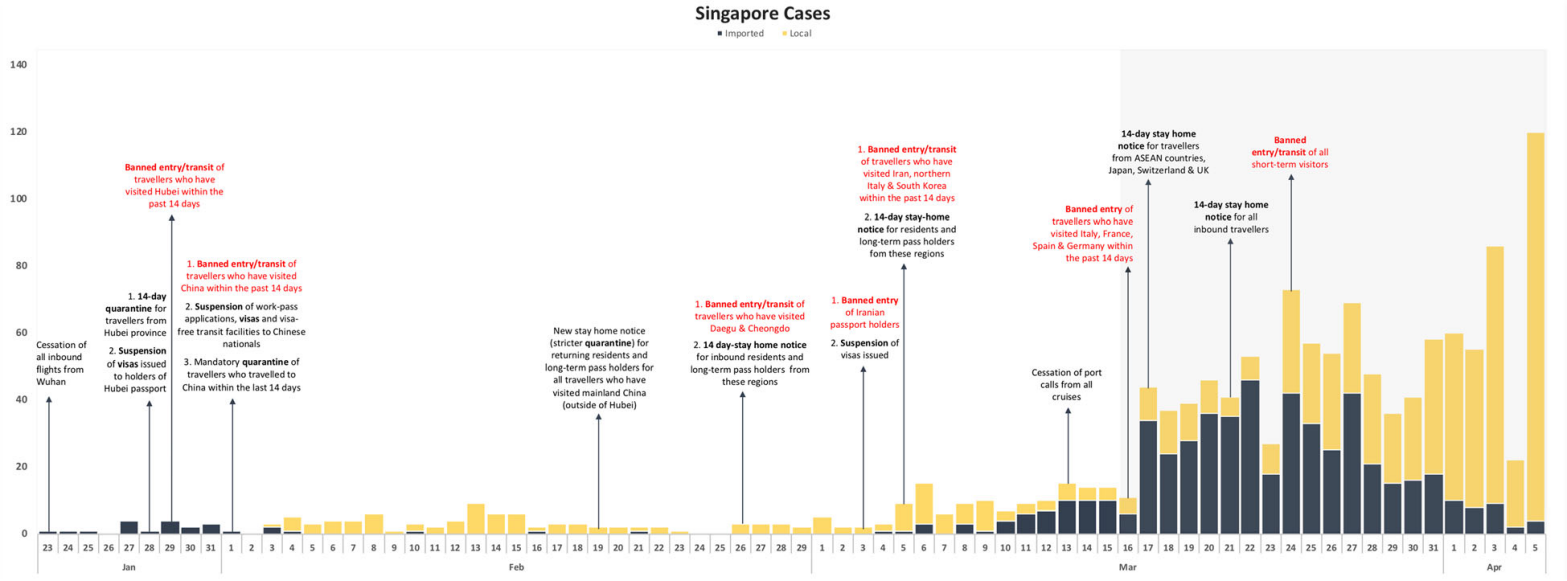
Epidemic curve of Singapore with interventions imposed (based on date of notification)

i. Singapore (Table 1 & Fig. 1)

**Fig. 2:**
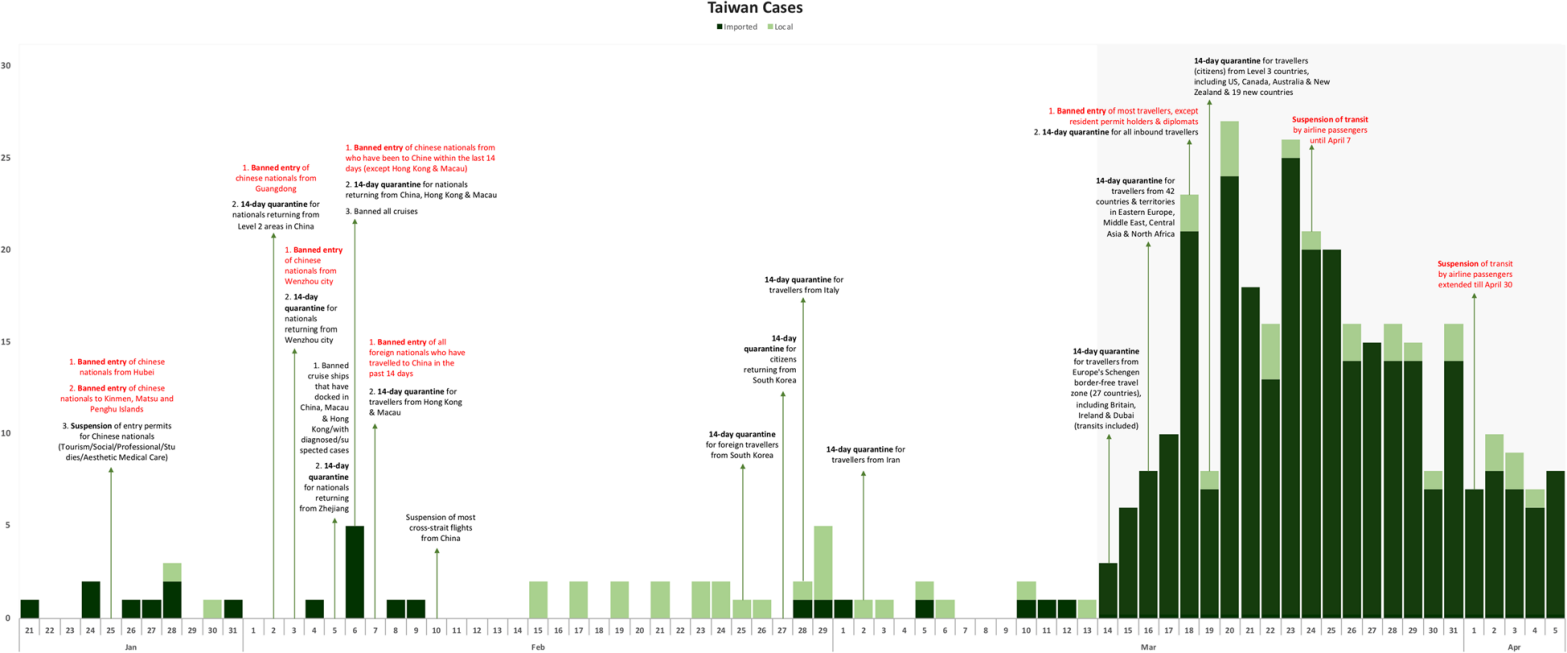
Epidemic curve of Taiwan with interventions imposed (based on date of notification)

Singapore first implemented its travel restriction against China by ceasing inbound flights from Wuhan on January 23, 2020. Over the next few days, measures were tightened with the introduction of 14-day quarantine, visa issuance suspension and banned entry/transit of travellers from Hubei. Despite having extended the travel ban to the entire China by February 1, Singapore recorded its first local case on February 3. New local cases were detected daily and declined after peaking on February 13, while imported cases were sporadically detected at low numbers. Local cases rose again during the period when travel restrictions were imposed on Daegu and Cheongdo, Iran and Italy. Imported cases re-surfaced on March 4 since the last case on February 21 and were consistently reported from March 8 onwards. Daily cases spiked on March 17, a day after Singapore imposed its first travel restriction against Europe. Between March 10 and 25, imported cases constituted most daily new cases. Local cases predominated after the implementation of Singapore’s ban on entry and transit of all short-term visitors.

ii. Taiwan (Table 1 & Fig. 2)

**Fig. 3:**
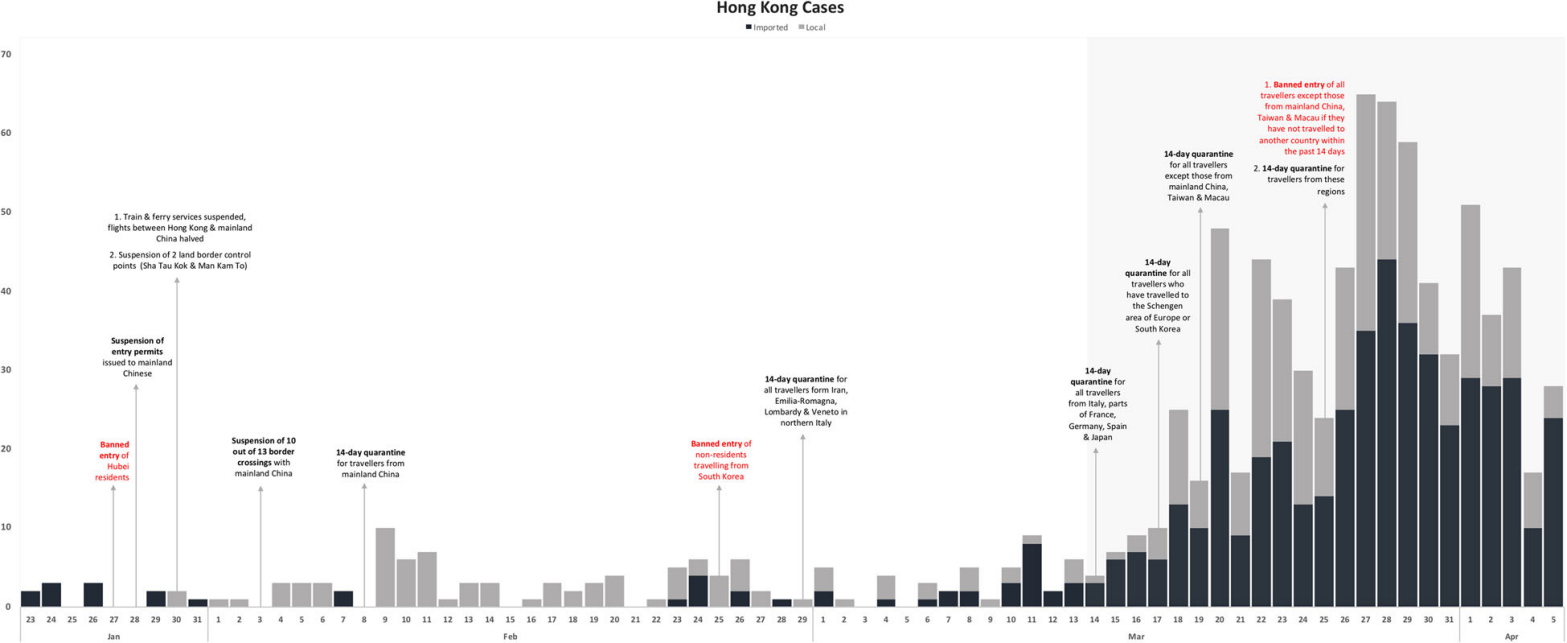
Epidemic curve of Hong Kong with interventions imposed (based on date of notification)

Taiwan had its first imported case on January 21 and introduced its first travel restrictions – banning entry of Hubei residents/Chinese nationals and suspending entry permits for Chinese nationals – on January 25. The first local case was reported 3 days later on January 28 and subsequently on January 30. Within the next week, Taiwan banned entry of Chinese nationals and introduced 14-days quarantine for returning citizens from China, Hong Kong and Macau. Notably, there were no local cases reported between January 30 and February 15. The subsequent period whereby 14-day quarantine extended to South Korea, Italy and Iran was marked by sporadic local cases. On March 14, the daily cases started to increase as travel restriction expanded to Europe and other regions, and with increased intensity from quarantine to banned entry and transit suspension. Local case count remained low during this period.

iii. Hong Kong (Table 1 & Fig. 3)

**Fig. 4:**
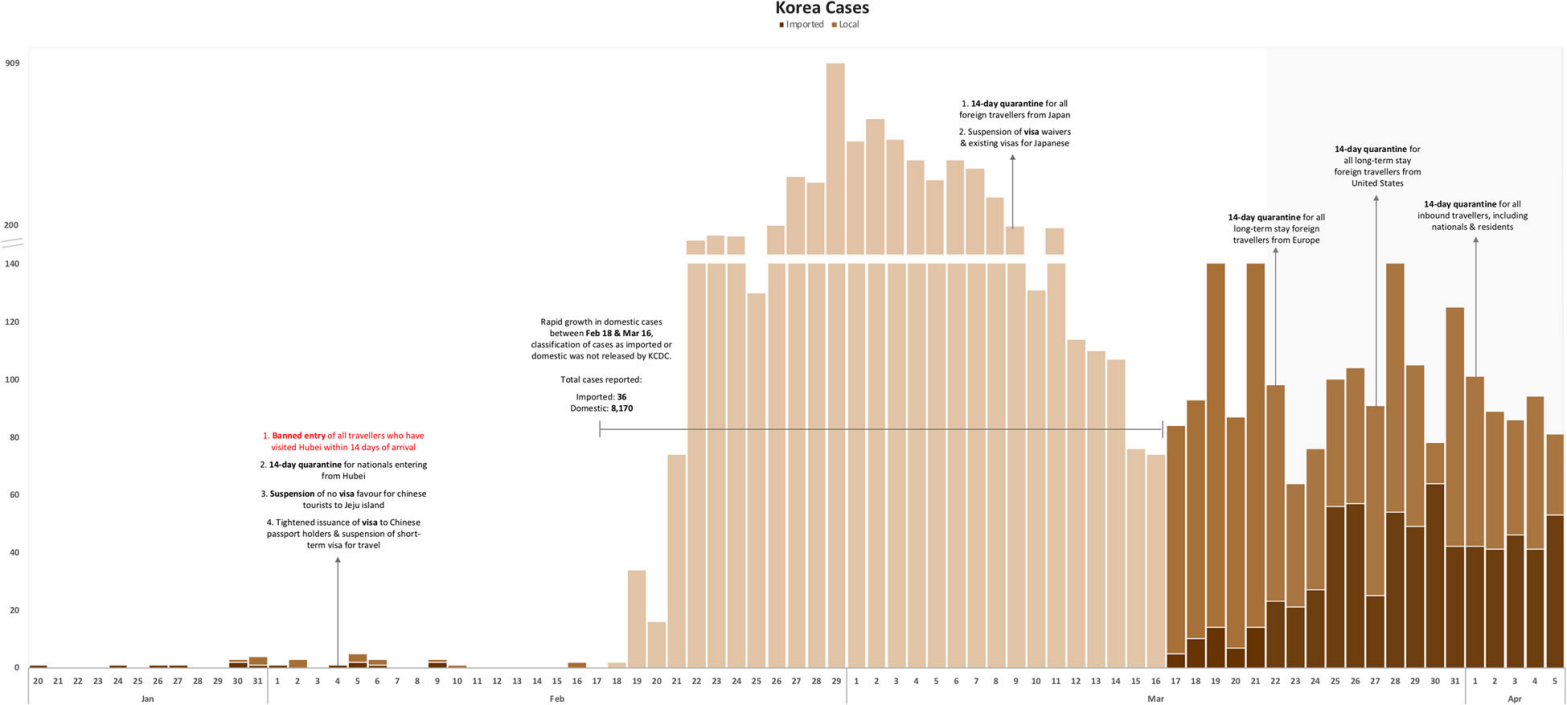
Epidemic curve of South Korea with interventions imposed (based on date of notification)

Hong Kong had its first imported case on January 23 and first banned entry of Hubei residents on January 27. The restriction was extended to include suspension of entry permit for mainland Chinese before the first local case was reported on January 30. Subsequently, border crossings between mainland China and Hong Kong were suspended and a 14-day quarantine was required of travellers. Local cases continued to occur in small clusters. Between February 25 and 29, Hong Kong banned entry of non-residents travelling from South Korea and introduced 14-day quarantine for Iran, parts of northern Italy. Local cases remained low and sporadic during this period. From March 14, the quarantine expanded to Europe and other countries as local cases started to increase. On March 25, a travel ban was placed on all travellers except those from China, Taiwan and Macau with no travel history in the past 14 days. A 14-day quarantine was, however, required for this group. After the tightening of travel restriction, local cases increased for approximately 2 days before declining.

iv. South Korea (Table 1 & Fig. 4)

Korea encountered its first imported and local case on January 20 and 30 respectively. Between the detection of the index case and the introduction of its first travel restriction against China on February 4, there were sporadic and relatively low numbers of COVID-19 cases. However, the daily cases surged from February 19 with local outbreaks erupting in Daegu and Cheongdo. On March 9, quarantine and visa suspension were introduced for travellers originating from Japan, and by then, daily cases had generally decreased since end-February. Breakdown of daily local and imported cases were not reported through March 16. Daily cases started to rise from March 17 following infusion of imported cases, while local cases remain high. This was despite introducing a 14-day quarantine for travellers from Europe, US and eventually all travellers. By April 5, the number of imported cases constituted approximately half of the total daily cases reported.

Overall, COVID-19 was the best contained in Taiwan, with daily reported cases not exceeding 30, and local cases (both absolute and incidence) consistently maintained at the minimum. The local situation in Taiwan, Hong Kong and Korea went under control as cases headed for a downward trend. However, Korea was seeing a resurgence of imported cases, contrary to the other three countries, while Singapore was experiencing increased local transmission.

### Comparison of countries’ response against different waves of epidemic centres & impact on imported cases

i. China (Wave 1)

Singapore was the only country to implement a travel restriction (against Wuhan) on the day the first imported case was reported (on January 23). However, it was not till January 29 that a full entry and transit ban on travellers who have visited Hubei within the past 14 days was implemented. Taiwan, Hong Kong and Korea recorded 3, 8 and 8 imported cases respectively before implementing their first travel restriction against Hubei Province; 4, 4, and 15 days after reporting their first imported cases respectively. Korea implemented its first travel restriction on February 4, more than a week later than the last of the three countries. Notably, only South Korea reported local transmission (7 cases) before any travel restriction was implemented.

ii. South Korea/Iran/Italy (Waves 2 and 3)

Singapore, Taiwan and Hong Kong were relatively similar in the pace of implementing restrictions against each wave of epidemic. However, Singapore lagged slightly after the first wave of actions taken against China. In the second wave of response against South Korea’s epidemic, Singapore implemented its first restriction two days after a state of emergency was declared in Korea, while Taiwan and Hong Kong both took one day. In the third wave of response against epidemics in Iran and Italy, Singapore took 12 and 8 days to enact any form of restriction for travellers from the respective regions; Taiwan took 8 and 4 days, while Hong Kong took 9 and 5 days respectively. Additionally, Singapore responded to the local epidemics in Korea by only banning and quarantining travellers who have visited Daegu & Cheongdo, while Taiwan and Hong Kong’s restrictions applied to travellers from the entire Korea. It was only in early March that Singapore extended its ban to all South Korea orginated travellers, together with the restrictions circumventing the third wave of epidemics in Iran and Italy. Following the third wave, imported and local cases in Singapore consistently surpassed that of Taiwan and Hong Kong. South Korea did not implement any restrictions on Iran and Italy.

Interestingly, neither Singapore, Taiwan nor Hong Kong reported any imported cases from Korea and Iran. Singapore confirmed its first imported case from outside China on March 4, in a traveller who had been to Europe (France, Portugal and the United Kingdom). Since then until before the first Europe-related travel restriction was implemented, Singapore recorded 56 imported cases – 21 were epidemiologically linked to travel solely in Europe, and 4 others were linked to multiple countries including Europe. Taiwan confirmed its equivalent on February 6, in a traveller who had been to Hong Kong and Italy. From then until before its first Europe-related restriction, Taiwan recorded 12 imported cases, only 2 were epidemiologically linked exclusively to Europe, and 5 others were linked to multiple countries including Europe. Hong Kong’s first non-China imported case, apart from those disembarked from cruise, was confirmed on March 4 in a traveller who had been to India. Before its first Europe-related restriction, Hong Kong received 22 imported cases – 4 were epidemiologically linked exclusively to Europe, and 1 was linked to multiple countries including Europe. Notably, imported cases from the United States surfaced even before travel restrictions targeted Europe’s emergence as the epicenter. In the same period, 10 of 56 imported cases in Singapore were linked to the United States. There was 1 case in Hong Kong who had travelled to US amongst other countries, while Taiwan recorded none.

iii. Europe, excluding Italy (Wave 4)

As the COVID-19 epicenter shifted to Europe in mid-March, Taiwan and Hong Kong swiftly implemented 14-day quarantines on the day Europe was declared an epicenter. Taiwan’s quarantine was more extensive and applied to all European travellers from the Schengen border-free zone. Singapore followed 2 days after, implementing a ban on inbound visitors from 4 European countries. South Korea once again, implemented its restriction – a 14-day quarantine – almost a week belatedly, on March 22.

Following the implementation of Europe-targeted restrictions, cases continued in an upward trend in all four countries for at least 7–15 days before peaking, with Singapore recording its maximum imported cases first within 7 days (Figs. 1, 2, 3 and 4). Moving average of daily imported cases decreased towards late March for Singapore, Hong Kong and Taiwan (Fig. 7, Supplementary Figs. S1-S9). A comparison of weekly imported cases revealed that cases peaked at week 12 for Singapore, and week 13 for Taiwan, Hong Kong and South Korea. Although South Korea recorded the most imported cases, incidence per 100,000 stood at a mere 0.62, compared to 3.66 cases per 100,000 during Singapore’s peak (Fig. 5, Supplementary Figs. S10-S13).

**Fig. 5:**
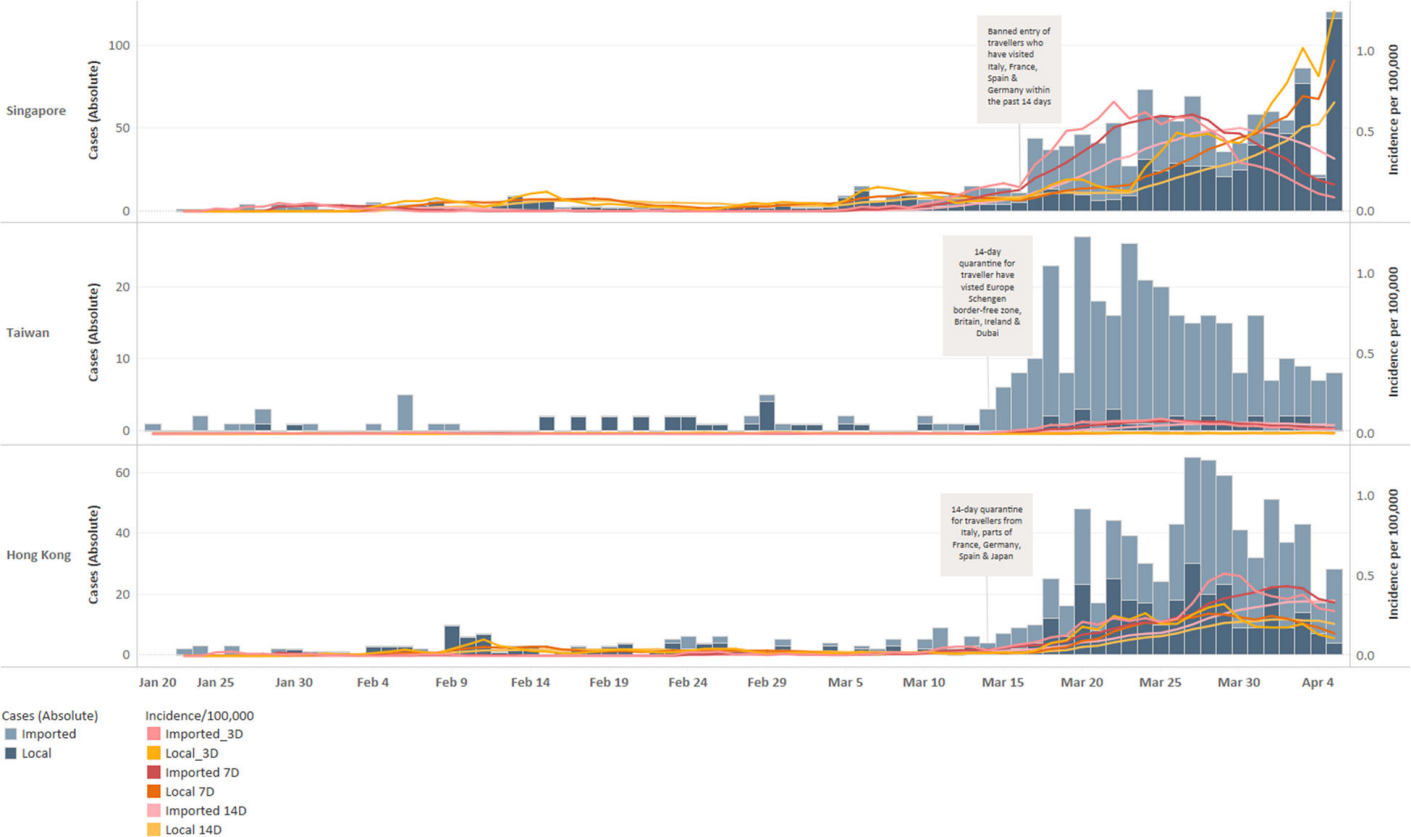
Comparison of daily new imported and local cases (3/7/14-day moving average)

**Fig. 6:**
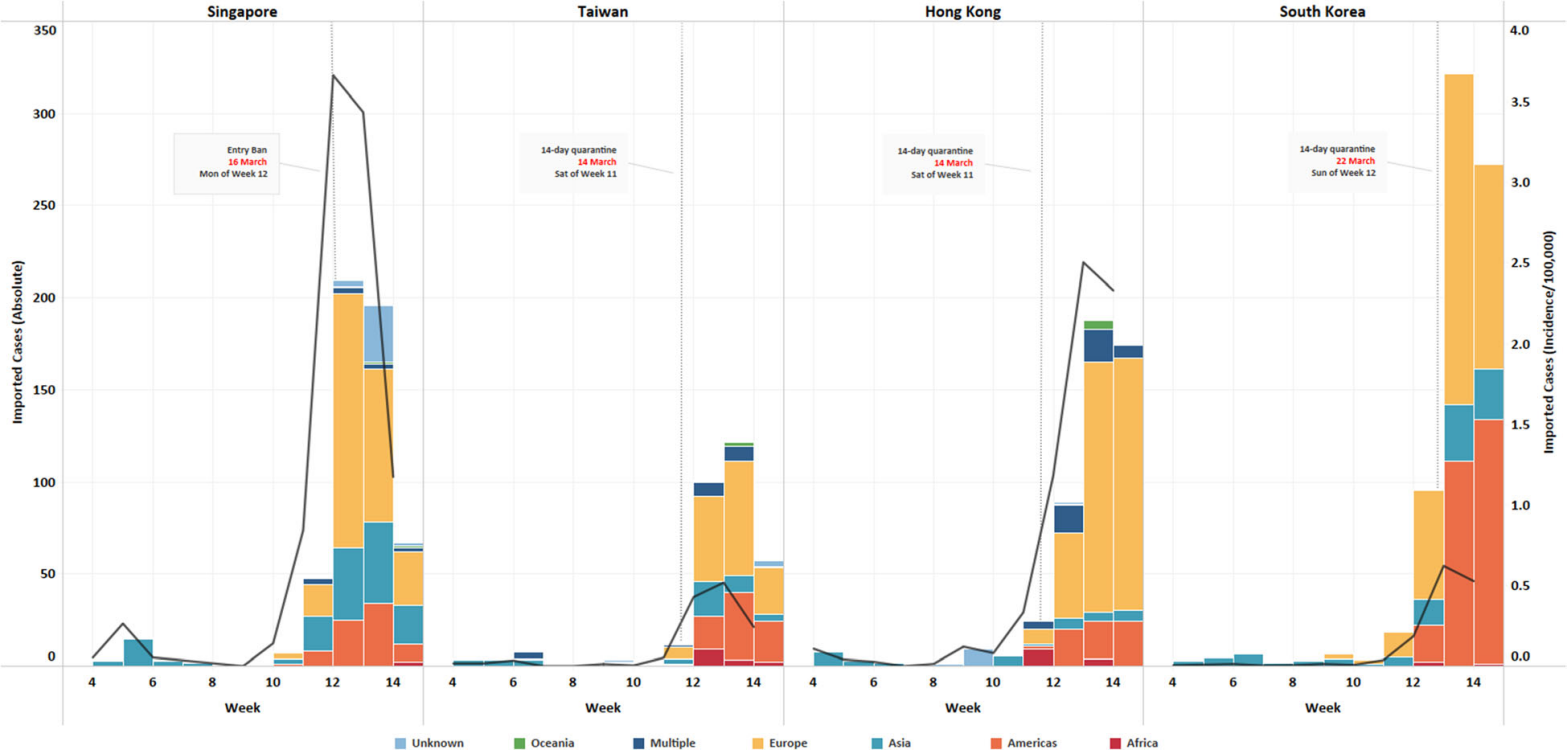
Comparison of absolute number (bar graph; primary y-axis, on left) and incidence per 100,000 (trendline; secondary y-axis, on right) of imported cases, by weeks in each country with the first Europe-targeted travel restriction highlighted

Europe was the greatest source of imported cases for all four countries (Fig. 6, Supplementary Fig. S14). All countries detected cases from Europe (week 9–10) even before travel restrictions were implemented against the region. The proportion of cases traced to Europe peaked in week 12, shortly after travel restrictions targeted the region. Notably, Taiwan received much less imported cases from Europe compared to the other countries, despite relatively similar proportions imported from other regions. Cases returning from the Americas were evidently on the rise starting from week 12, before dropping in week 14 for Singapore and Taiwan, and plateauing for Hong Kong.

iv. All countries (Wave 5)

The pattern in speed of issuing travel restrictions by all four countries were consistent across all waves. In terms of a sweeping ban against inbound travellers, Taiwan acted as early as March 18, way ahead of Singapore and Hong Kong. Singapore, Taiwan and Hong Kong took actions against all international travellers 80, 77, and 78 days respectively, after the outbreak began in Wuhan. By March 25, entry of most international travellers was prohibited with few exceptions. Korea only implemented its equivalent, a 14-day quarantine on all inbound travellers in early April – 91 days after outbreak began in Wuhan.

### Rate of change and correlation among imported and local cases

**Fig. 7:**
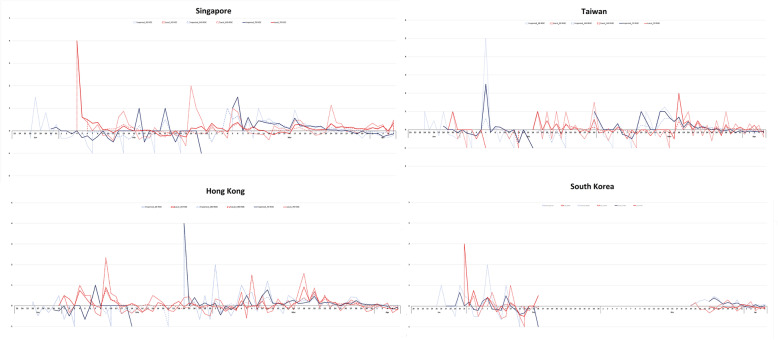
Comparison of rate of change of imported and local cases (3/7/14-day moving average; 7 as main, 3/14 as reference)

The rate of change of imported cases was similar across all four countries – characterized by a fall from late January to early/mid-February before rising, a subsequent period of sporadic importation during late February, and a final decline to the negatives after a rise in March (Fig. 7). From the time of the first wave of restrictions until before the second wave of restrictions were implemented, the rate of change of imported cases in Singapore, Taiwan, Hong Kong and South Korea (until February 16) fell by 1.08, 1.2, 1.2 and 1.4 respectively. While Singapore banned travellers who have been to China 14 days prior to entry, cases from China remained the main source of importation before March 4. A fall in rate of imported cases was observed only until mid-February. This suggests that preventing importation of cases from epidemic regions through travel restrictions was effective only for a short duration before scattered cases from the epidemic regions streamed in from other regions. Following the final wave of restrictions, the rate of change of imported cases fell by 0.35, 0.22 and 0.52 respectively for Singapore, Taiwan and Hong Kong. The rate of change in local cases across all 4 countries showed unremarkable differences in trajectories in the early stages. From March 18, a consistent decrease in rate of change below 0 was observed for Taiwan (2 to − 0.111) and Hong Kong (0.917 to − 0.204). Korea saw its rate largely in the negatives range from March 24 (− 0.0475 and − 0.0793). Singapore, however, maintained a positive rate of change in local cases (0.259 to 0.339) since March 17.

No consistent trend was observed in the rate of change of local cases following each wave of intervention against the emergence of local epidemics (Supplementary Figs. S15–17). A decreasing rate of change could only be observed in our analysis of 14 days after the final wave of interventions. No discernable correlation was observed between imported and local cases in all four countries following each wave of intervention (Supplementary Tables S1-S6). Utilizing rate of change calculated (Supplementary Tables S7-S12) using 7-day moving average of imported and local cases, we found the correlation coefficient was largely positive but weak, with most ranging between − 0.5 and 0.5 for both 7 and 14 days post each wave of intervention (Supplementary Tables 3&4). This lack of consistent positive correlation suggests that our study did not find any delay in rate of change of local cases as a result of travel ban.

## Discussion

### Importation risk of the countries

The risk of importation from China largely diminished after the initial stages. Despite local epidemics in various countries, the largest risk of importation stemmed from Europe, as cases with related travel history emerged as early as two weeks before Europe was declared an epicenter. Singapore received the most cases epidemiologically linked to Europe before any restriction targeted the region. While cases from Europe peaked for all countries in early April, it is evident that the United States was an emerging region of risk from week 12.

The marked lag in South Korea’s first travel restriction coincides with its status as the first country outside China to face a large-scale local COVID-19 epidemic. Notably, it was the earliest to detect an imported case, and the only country to record local cases before any action was taken against travellers. This suggests that COVID-19 could be more widespread in Korea even at the early phase due to the headstart in arrival and delayed implementation of any travel restriction. Overall, its highest importation of cases may be attributed to its consistent imposition of travel restrictions belatedly. Korea’s relatively high pre-pandemic air traffic was a possible explanation for its highest importation of cases (in terms of absolute numbers) during the early stages of the pandemic. While the countries enjoy similar status as global air hubs, differences in air traffic during the pandemic period could be attributed to varying overseas populations, displaced for work or academic purposes.

With a population of less than 6 million, Singapore saw the greatest incidence of imported cases per 100,000 population. This could be due to two mechanisms. Singapore’s risk of importation was inherently higher than Taiwan and Hong Kong with its greater passenger arrivals in January to March. Singapore continued to receive high numbers of inbound travellers during the early pandemic stages (828,317 in March 2020) as compared to 213,710 and 349,248 arrivals in Taiwan and Hong Kong [33–35] despite having lower passenger arrivals than Hong Kong in pre-pandemic times. Coupled with its highest population density among the Asian countries, the high incidence of importation increases risk of outbreak occurrence. Moreover, despite being the most prominent in dishing out travel bans as opposed to quarantines, Singapore’s bans against each wave of potential importation were slower than Taiwan and Hong Kong. This coincides with an observed increase in Singapore’s local cases as of early April 2020.

### Effect of travel restriction on imported cases

While an overall reduction in rate of change of imported cases was observed for all four countries after the first and final wave of restriction imposition, the persistent rise in imported cases following the Europe-targeted travel restriction suggests the ineffectiveness of travel bans once epidemics become widespread.

The effectiveness of travel ban hinges on timeliness and extensiveness of implementation. While there is consensus that Wuhan’s lockdown on January 23 substantially averted exportation of cases globally [58, 59], Chinazzi et al. (2020) found that the epidemic had already become widespread in other mainland Chinese cities by then [60]. Yet, the first wave of restrictions against imported cases from China were only implemented between January 28 and February 4. In particular, the Europe-related travel restrictions came undoubtedly late for all. Singapore, Taiwan and Hong Kong respectively recorded imported cases from Europe two weeks before any restriction targeted the region. Phylogenetic analysis of virus strains in Americans traced primarily to Europe since February [61]. This suggests transmission of COVID-19 in European communities even before outbreaks were reported. Like the initial lockdown in Wuhan, restrictions targeted at Europe were imposed only after Europe originated travellers have likely seeded epidemics elsewhere. Constantino et al. (2020) demonstrated that travel restrictions could be effective provided there is only a single source of exportation [62], often the case only in early stages of an outbreak. Effectiveness diminishes as the outbreak becomes more widespread in the current climate of global connectivity. Substantial evidence suggests the need of a near complete shutdown of global travel to contain the epidemic modestly [6–9].

There are many reasons why a travel ban can come too late. The International Health Regulations (2005) binding 196 countries states that measures to address public health risks should be grounded by WHO advice and prohibits implementation of additional health measures exclusively as a precaution. However, a Public Health Emergency of International Concern (PHEIC) was only declared on January 30, a month after news of an outbreak of pneumonia cases in Wuhan broke [63]. While countries look to the WHO in disseminating timely advice, sparse knowledge and the lack of pharmaceutical defense against the novel virus led to belated implementation of travel restrictions. Countries may also be cautious in implementing restrictions to preserve diplomatic relations with affected regions [64–66].

Public response and supplemental policies could undermine the effectiveness of travel restrictions. The imposition of a travel ban invokes heightened risk perception of the banned region. Given the exemption of nationals, residents and dependents from travel bans, their return from disease-affected areas translates to an influx of disease carriers into their home countries. Findings show that the Europe travel ban implemented by the Trump administration caused the final infusion of COVID-19 cases in America as an estimated 15,000 more Americans who had high risk of exposure returned following the announcement [67]. Singapore noted persistently high numbers of imported cases despite its first Europe-related travel ban on March 16. WHO’s declaration of COVID-19 a pandemic and Europe its new epicenter on March 11 and 14 fueled a spike in risk sentiment towards the region. On March 15, the Ministry of Education in Singapore recalled students on overseas placement and internships [68]. A 14-day stay-home notice (equivalent to a quarantine) was also mandated for those returning from ASEAN countries, Japan, Switzerland and the United Kingdom [69], causing travellers to swarm back in avoidance of quarantine. On March 17, the Ministry of Foreign Affairs further recalled Singaporeans studying abroad. These events collectively underlie the persistent rise in imported cases after March 16, driving up the incidence of imported cases in Singapore. Although we did not find similar recall advisories from Taiwan and Hong Kong, imported cases in Hong Kong towards late March largely constituted students who had been studying overseas, indicating a similar demographic among returnees. As states are responsible for their citizens abroad, governments may also evacuate citizens stranded in epicenters. Singapore conducted repatriations from Wuhan [70], Iran, UK, Cambodia, Egypt, India and Nepal [71–76]. Taiwan evacuated its citizens on at least 3 occasions between February 4 and March 29 while Hong Kong had repatriated 533 evacuees from Wuhan between March 4 and 5 [77]. South Korea deployed at least 3 chartered flights to bring back 848 evacuees from Wuhan between January 30 and February 11 [78, 79], with additional repatriations in late March and early April from Germany, Italy, Nepal and India [80]. This concurrent occurrence of multiple policies interplaying with public responses highlight the importance of strict and efficient arrival quarantine following travel bans to prevent spread to the community [81, 82].

### Other travel-related measures

Airport screening is commonly the first line of defense in border surveillance. While Singapore, Hong Kong and Taiwan supplemented it with travel bans and quarantines throughout the course of the pandemic, South Korea employed it as its main mode of defense for the longest period. Its special entry procedure encompassed a health and travel declaration alongside fever screening as travellers were also required to download a smartphone application to report their health condition over 14 days [83]. However, asymptomatic carriers or mild presentation of symptoms in infected individuals [84] limits the effectiveness of symptomatic border screening. Through April 6, less than half of imported cases in Taiwan developed febrile illness over the course of disease [85] while 90% of imported cases in Singapore over three days in March did not show symptoms upon arrival [86]. A recent study attributed undocumented infections as the source of 79% of documented cases [87], indicating that a reduction of undocumented infections through active case finding could help with the control and curb COVID-19 spread. The presence of undocumented imported cases is extremely perilous during the early stages of the epidemic as it introduces the virus to a naïve population. This study could not discern if higher number of cases imported to South Korea in the absence of quarantine before March 22 manifested in greater secondary local transmission as we did not find consistent positive correlation between rate of change in imported and local cases. However, Hossain, et al. (2020) found that even a full-scale border control without proper quarantine measures will only have limited effect if epidemic growth at the source is already high [82]. Drawing from Taiwan’s data, 67.3% of imported cases from January 21 to April 6 undetected during airport screening went on to infect 19 individuals, constituting 36.5% of local infections [85]. Enforced physical distancing through quarantine is, hence, essential in supplementing border control measures that may miss asymptomatic travellers, those incubating the disease or purposely concealing their fever [88].

### Effect of travel restriction on local cases

No obvious correlation was detected between the rate of change in imported and local cases. Correlation analysis of the rate of change in imported and local cases following all waves of restriction was unremarkable – relationship between imported and local cases were largely positive but weak for all countries. A decreasing trend was only observed in the rate of change of local cases following the final wave of restriction, more prominent in the 14-day post-intervention window than 7 days. However, this decreasing trend may also be attributed by an extensive push for social distancing in the respective countries. Most of the community distancing measures undertaken were relatively matched in pace [89–91]. Closure of most entertainment venues started in late March. While stricter distancing rules were imposed in public spaces in Singapore, the equivalent was disseminated as advice in Hong Kong, Taiwan and South Korea. Regardless, the persistent rise in daily local cases in Singapore prompted a circuit breaker, the country’s equivalent of a soft lockdown, on April 7.

Hong Kong and Taiwan’s success with containing COVID-19 stems from a holistic integration of travel related and local control measures. Local cases have remained sporadic throughout the early stages of the epidemic even without denying entry to travellers, suggesting that arrival quarantines are sufficient to prevent spread to the community when complemented by robust local control measures. Enhanced laboratory testing of influenza-negative patients with severe respiratory symptoms doubled as effective sentinel surveillance for COVID-19 in the community. Active COVID-19 testing was extended to patients with severe respiratory symptoms in the community in Taiwan and Hong Kong from mid-February, and Singapore as early as late January [92–95]. From March 19, Hong Kong made it compulsory for asymptomatic individuals under mandatory quarantine to undergo COVID-19 swab tests. Taiwan and Singapore, on the other hand, retrospectively tested symptomatic cases. South Korea only started testing all European arrivals for COVID-19 since March 22. Active case-finding and contact tracing are crucial in epidemic control. Despite the different approaches in COVID-19 testing, all four countries were reputed to have well-utilized widespread testing and/or aggressive contact tracing using data analytics. In South Korea, testing was accessible to the public as test kits and drive-through checkpoints were available from February. Digitalized contact tracing and transparency in information disclosure constitutes the core of Korea’s containment efforts [96], while Taiwan integrated its national health insurance database with immigrations and customs data to aid in rapid case identification [97]. For Singapore, contact tracing is an elaborate operation entailing multi-agency cooperation, surveillance footage and data visualization that was deemed gold standard by the WHO in early February.

### Limitations

There are a few limitations in this study. First, inconsistencies in reporting by the countries limited several aspects of the analysis. As Singapore did not report symptom onset date and South Korea reported neither symptom onset dates of cases nor individualized case data, cases in our analysis were based on confirmation date instead of symptom onset, a more accurate measure of epidemic progression. The analysis for South Korea was also constrained by missing data during the mid-February to March period when local epidemics broke out. Due to geographical and connectivity differences, the trend of imported cases observed cannot be generalised. In addition, imported cases were not normalized to travel volume affected by mounting travel restrictions at the borders due to the lack of publicly available data at the required granularity. This potentially hindered a more accurate quantification of true risk posed by COVID-19 importation, barring existing travel restrictions, into the four countries.

Surveillance bias in a healthcare system occurs when occurrence of disease is searched with differential intensity according to the healthcare setting and patient type [98]. Singapore, Taiwan and Hong Kong actively tested patients with severe respiratory symptoms in the community for COVID-19 since end January/mid-February. South Korea went further in having widespread publicly accessible testing facilities (drive through) since early February. Testing in the four countries differed in coverage. Additionally, testing in Singapore, Taiwan and Hong Kong is reactive, mostly government-mandated due to plausible exposure, while the South Korean population is actively encouraged and supported by the government to do so [99], contributing to early and greater detection. Our findings also cannot be extrapolated to countries with differential surveillance capacity and strategies.

Different health-seeking tendencies in communities may also impede detection of cases. It is possible that asymptomatic patients or those with mild condition may not seek medical treatment, hence, go undetected by the healthcare system [100]. Preliminary finding in Singapore suggests that COVID-19 patients were uncertain in whether to seek medical treatment as they did not have fever and symptoms were mild and non-specific [101]. Apart from the disease characteristics, cultural and systemic differences may influence health-seeking behavior of the population. Factors like low socioeconomic status, differing medical coverages and different cultural and beliefs can also deter communities from seeking help upon illness [102, 103]. Korea’s push for public’s uptake of testing, for example, is aligned with its population’s disposition towards seeking healthcare [104].

Lastly, the effectiveness of the measures implemented by the four countries may not be generalizable to other countries. These countries have a smaller population as compared to the larger western countries [105]. Differences in social structures and traditions may also challenge the implementation of non-pharmaceutical measures [106]. Moreover, the management of health systems differs across countries – smaller nations like Singapore and Taiwan have a unitary government that manage the healthcare for the whole nation [107], while larger countries, such as Canada has a federal system where there are 13 separate health jurisdiction that deliver different response to the pandemic. The Asian countries examined were also better poised to deal with outbreaks given their past pandemic experience.

## Conclusion

Travel bans and restrictions may be effective only in the initial stages to prevent virus importation into a naïve population. More well-designed studies are required to investigate travel restriction’s effect on local transmission. Apart from travel restrictions, multi-pronged approaches including social distancing measures, increased test capacity, prompt contact identification and isolation remain critical to control community transmission.

## Supplementary Information

**Additional file 1.**

## Data Availability

The datasets used and/or analysed during the current study are available from the corresponding author on reasonable request.
