# Supplementary material for: Impact of travel ban implementation on COVID-19 spread in Singapore, Taiwan, Hong Kong and South Korea during the early phase of the pandemic: a comparative study
Source: BMC Infect Dis. 2021 Aug 11;21:799. doi: 10.1186/s12879-021-06449-1 (PMC8355580; doi:10.1186/s12879-021-06449-1)
Supplement: Supplementary file 1 — Additional file 1. [file 12879_2021_6449_MOESM1_ESM.docx]

## I. Figures

#### Rate of Change


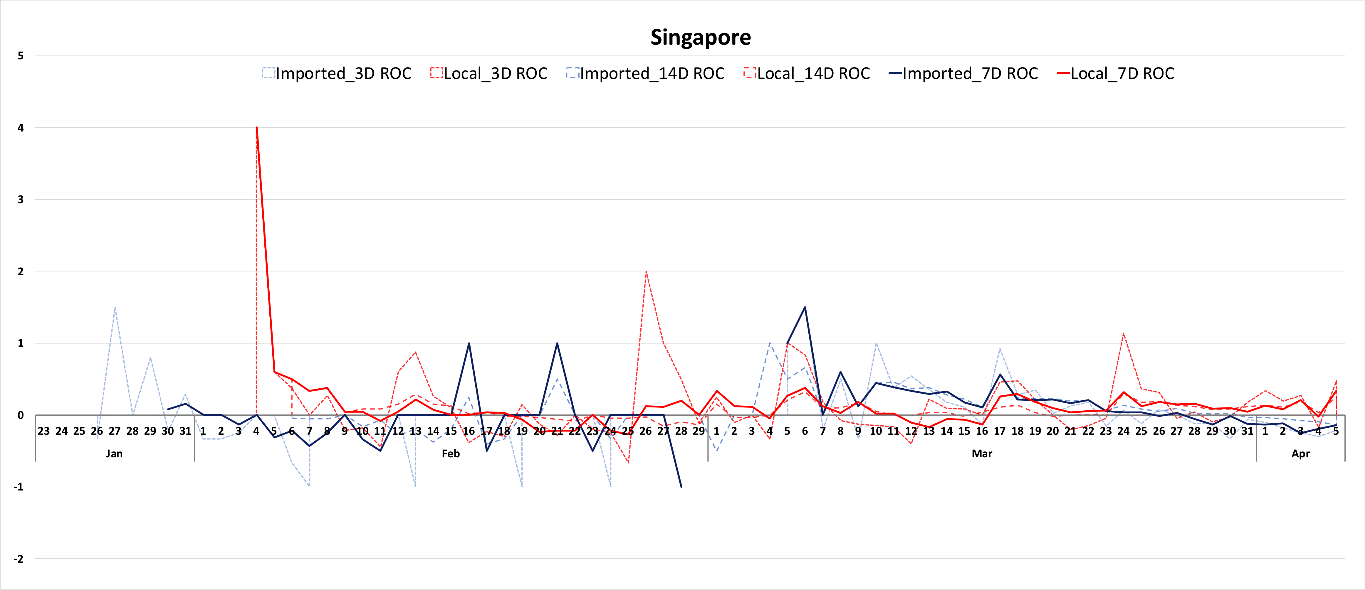


Figure S1: Rate of change of imported and local cases in Singapore (3/7/14-day moving average)


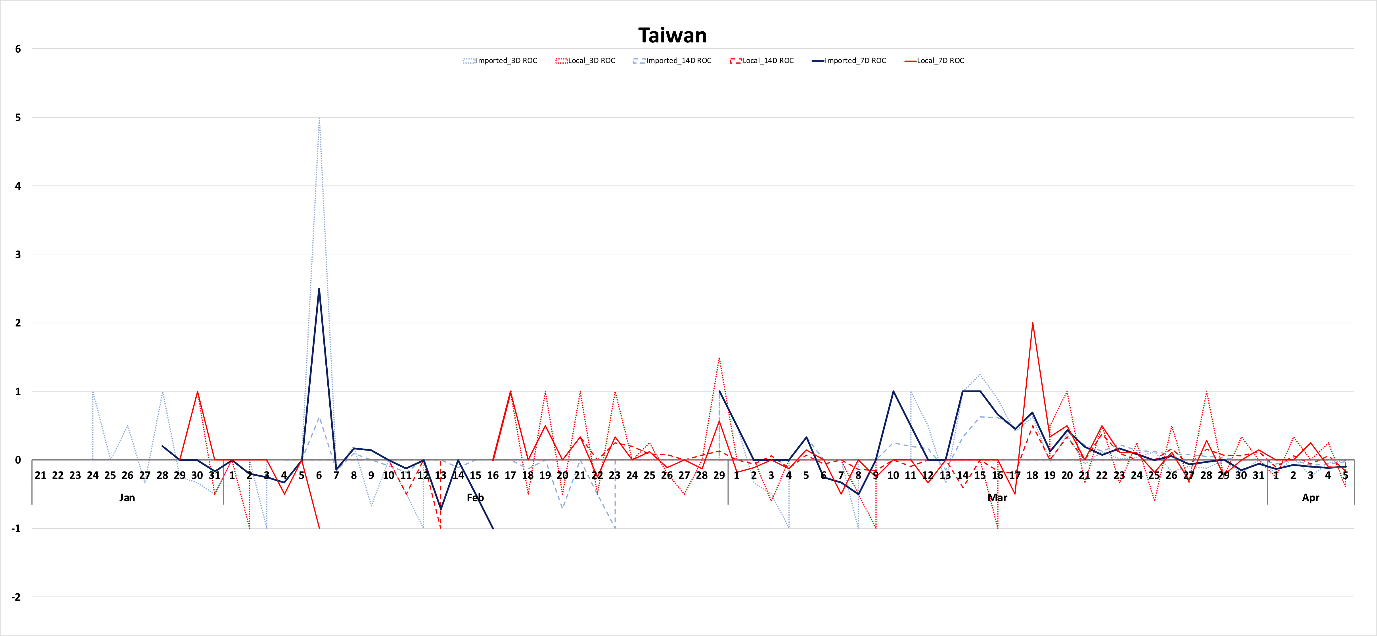


Figure S2: Rate of change in imported and local cases in Taiwan (3/7/14-day moving average)


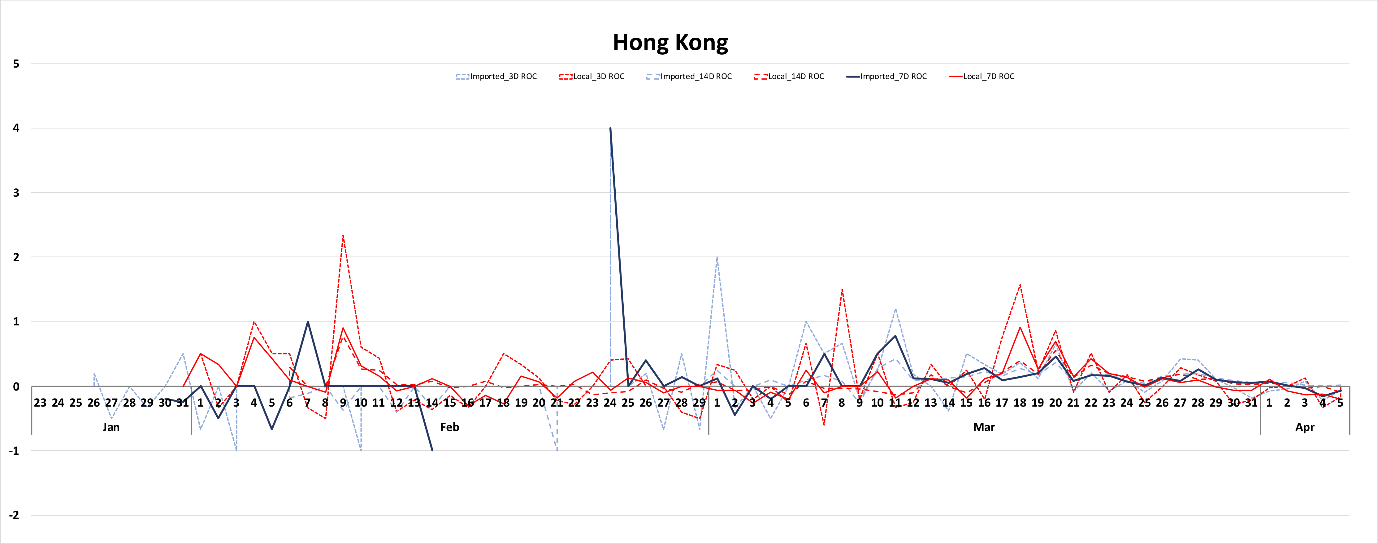


Figure S3: Rate of change in imported and local cases in Hong Kong (3/7/14-day moving average)


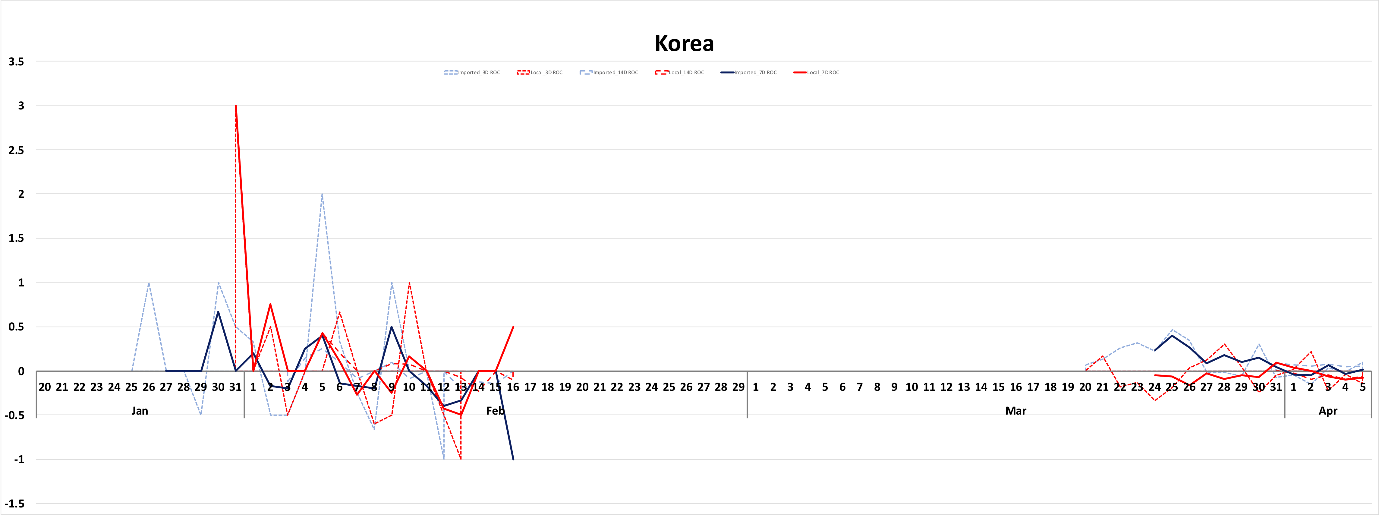


Figure S4: Rate of change in imported and local cases in Korea (3/7/14-day moving average)


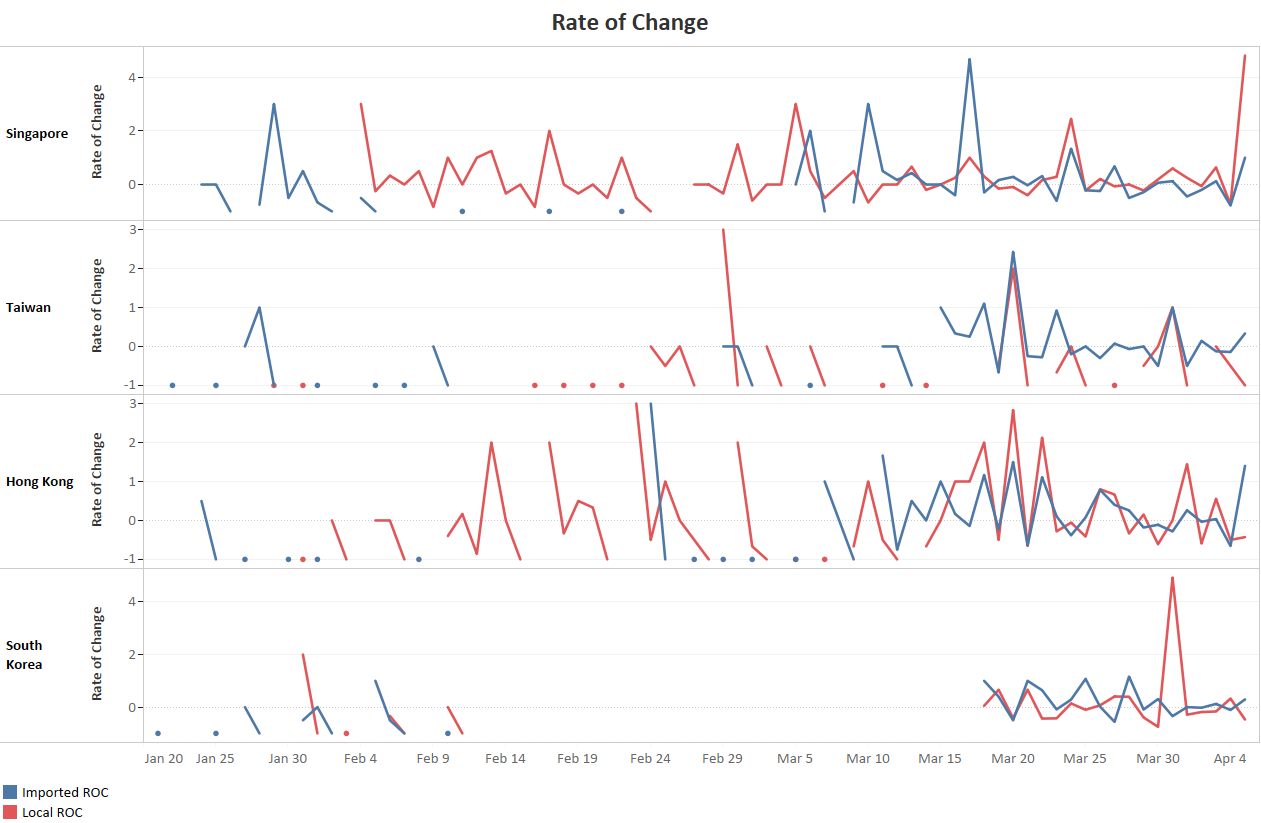


Figure S5: Comparison of rate of change of imported and local cases


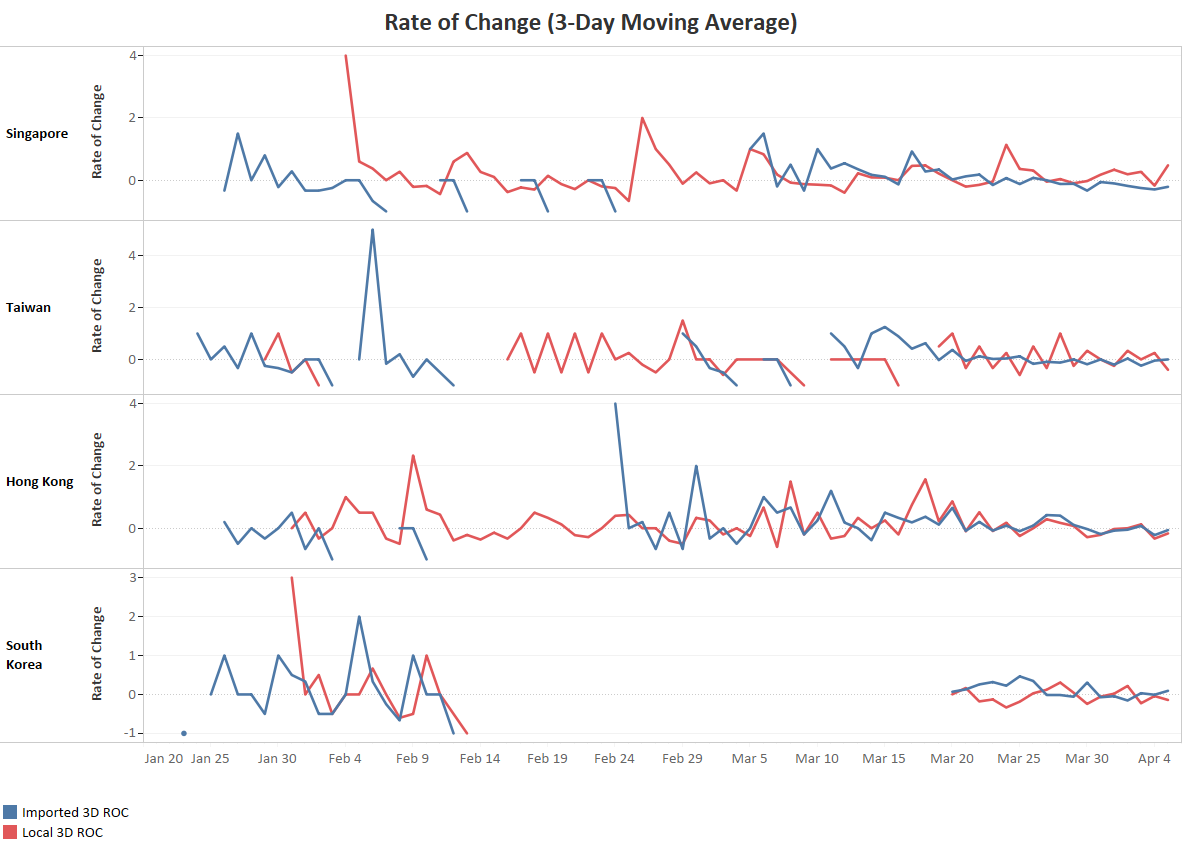


Figure S6: Comparison of rate of change of imported and local cases (3-day moving average)


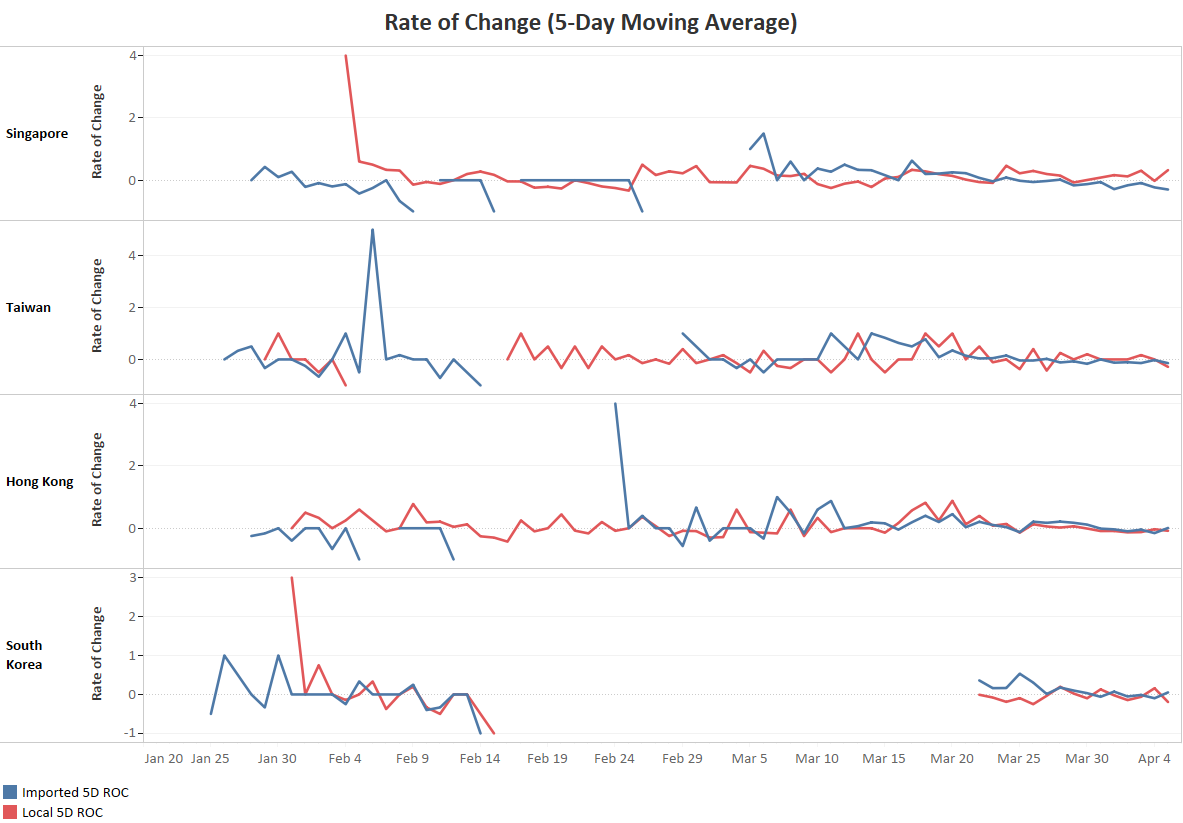


Figure S7: Comparison of rate of change of imported and local cases (5-day moving average)


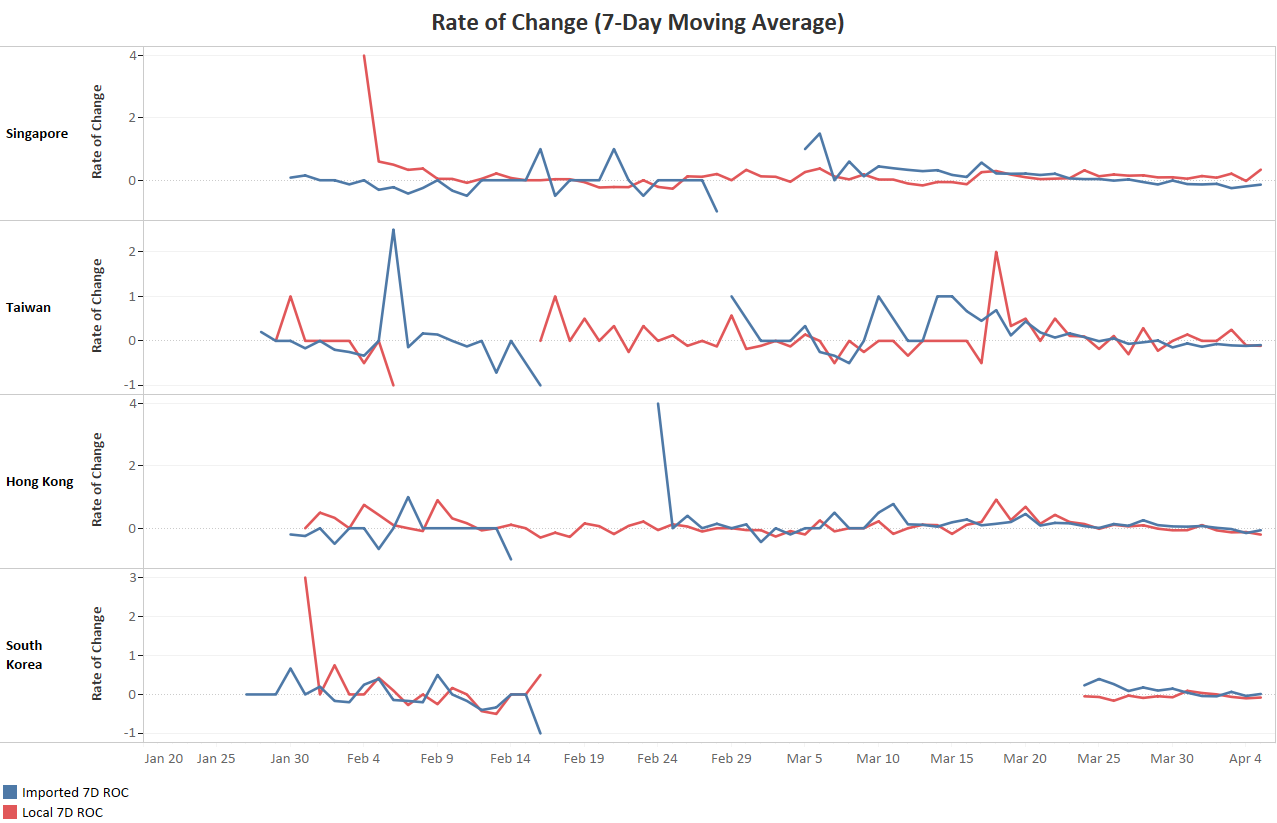


Figure S8: Comparison of rate of change of imported and local cases (7-day moving average)


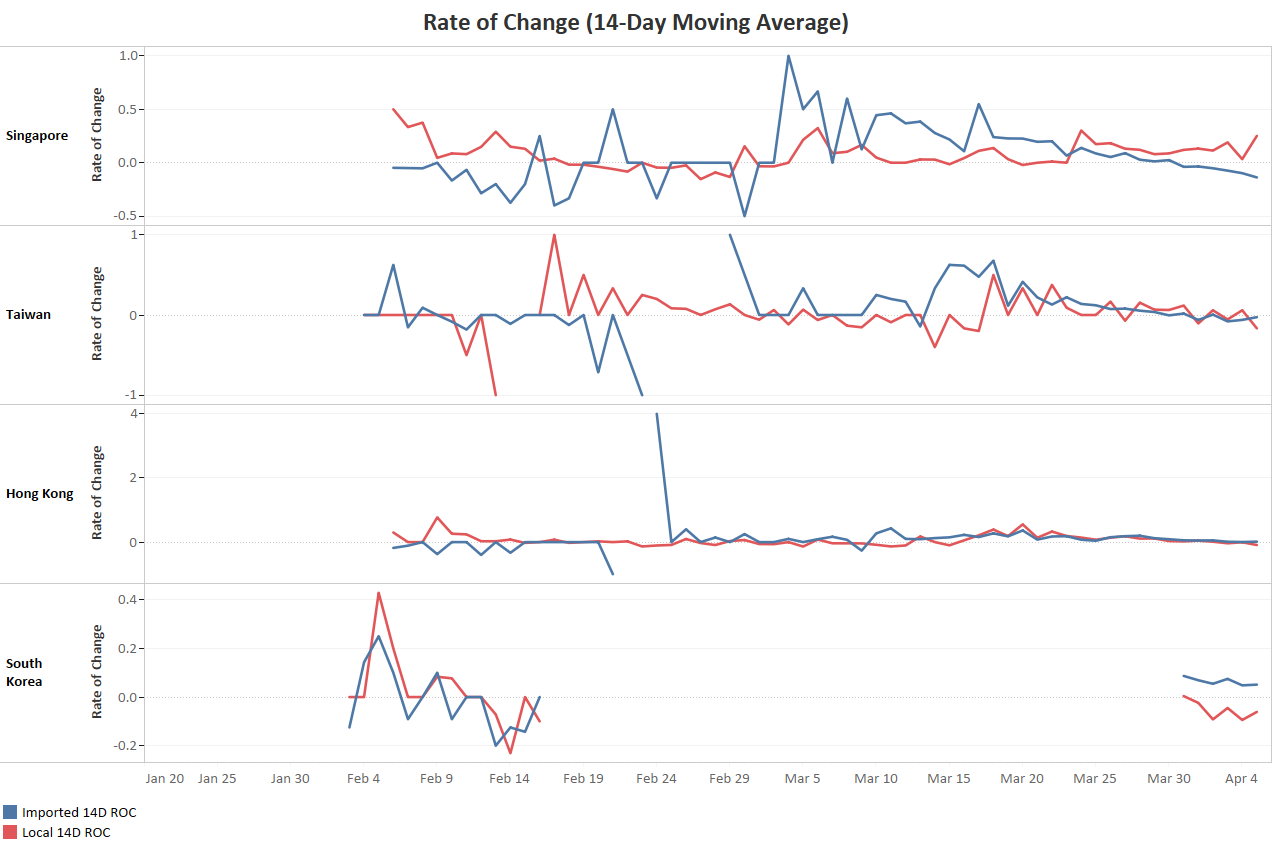


Figure S9: Comparison of rate of change of imported and local cases (14-day moving average)

#### Daily Cases (Moving Average)


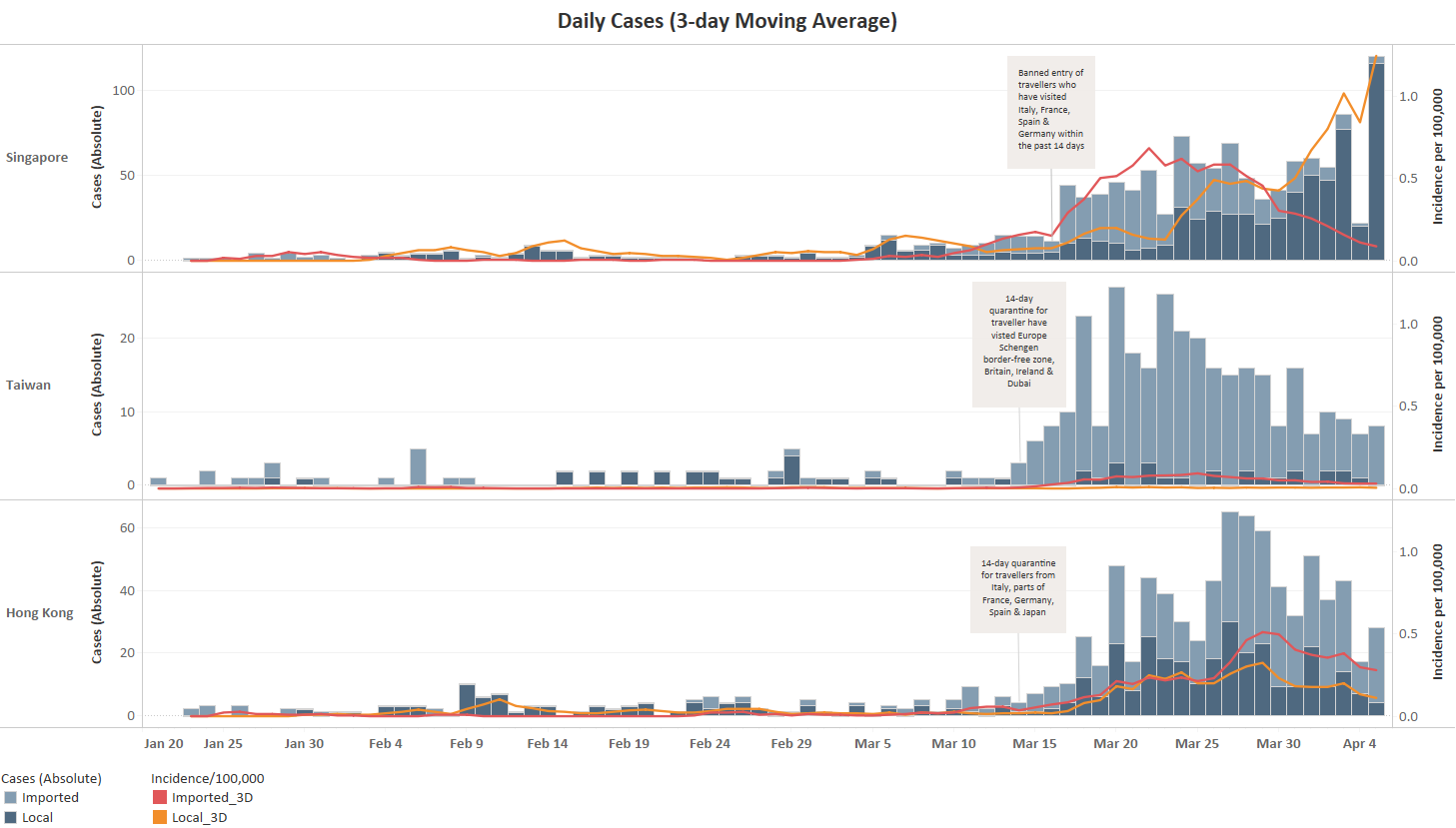


Figure S10: Daily new imported & local cases in Singapore, Taiwan & Hong Kong (3-day moving average; first Europe related restriction highlighted)


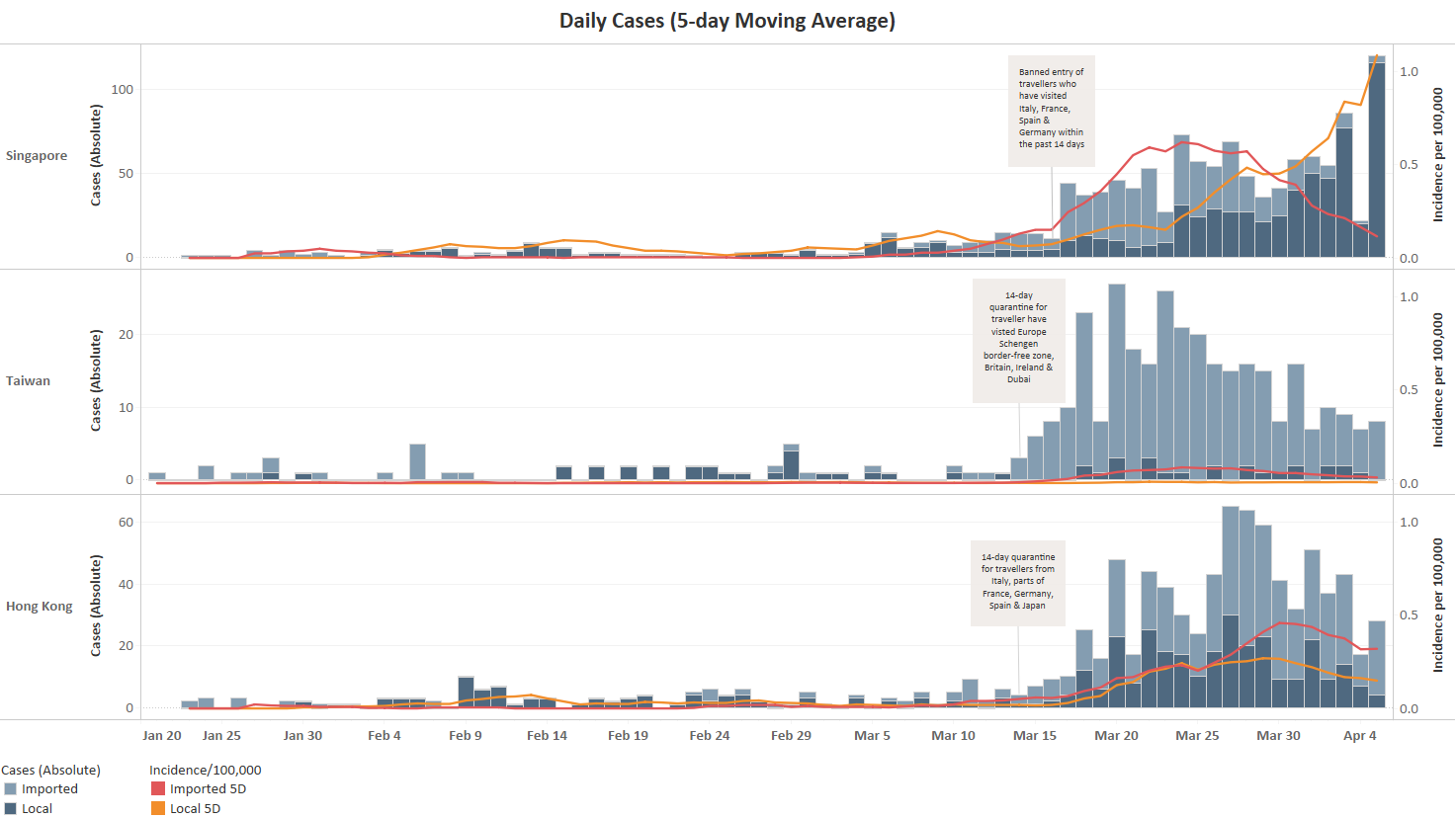


Figure S11: Daily new imported & local cases in Singapore, Taiwan & Hong Kong (5-day moving average; first Europe related restriction highlighted)


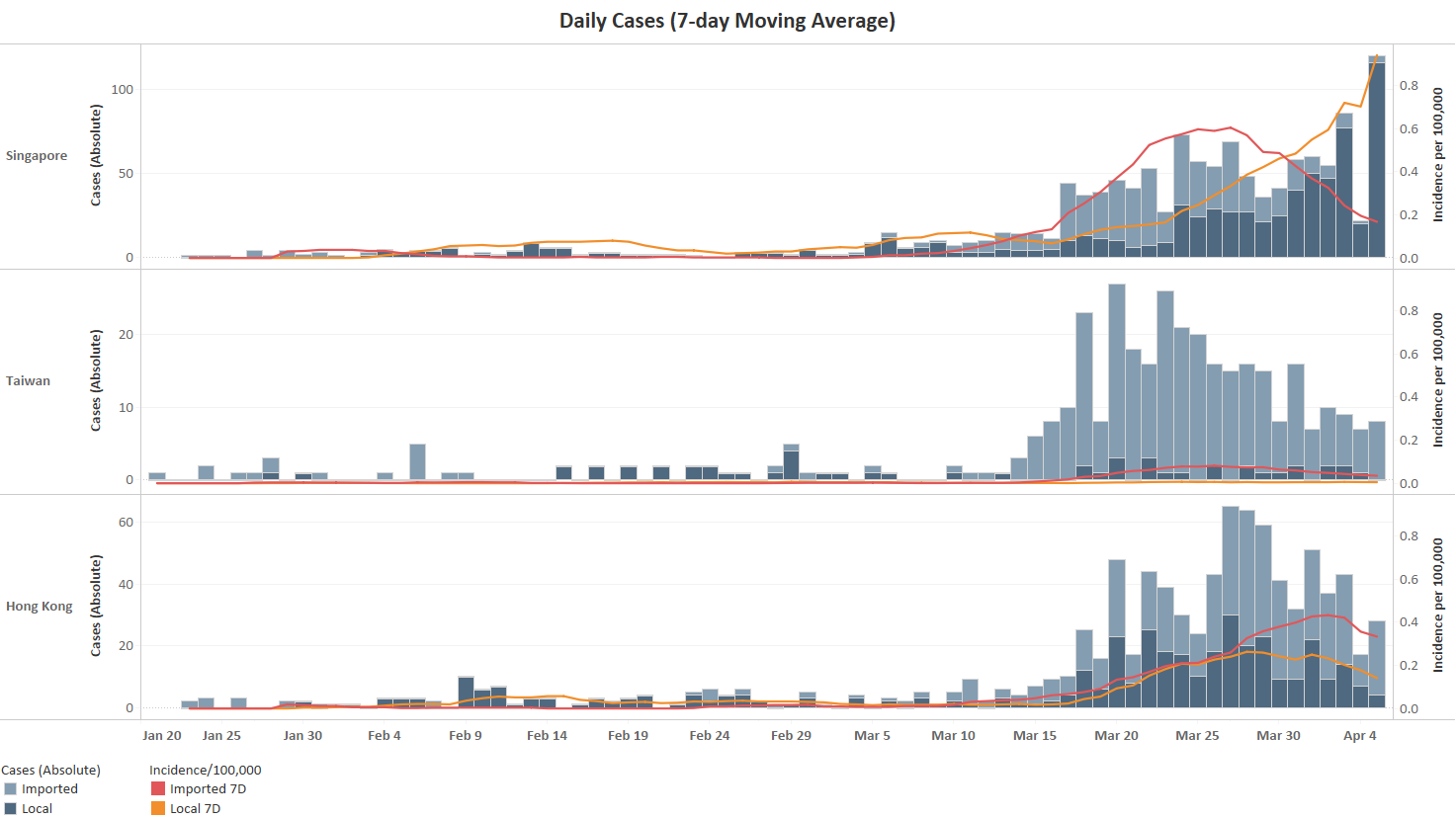


Figure S12: Daily new imported & local cases in Singapore, Taiwan & Hong Kong (7-day moving average; first Europe related restriction highlighted)


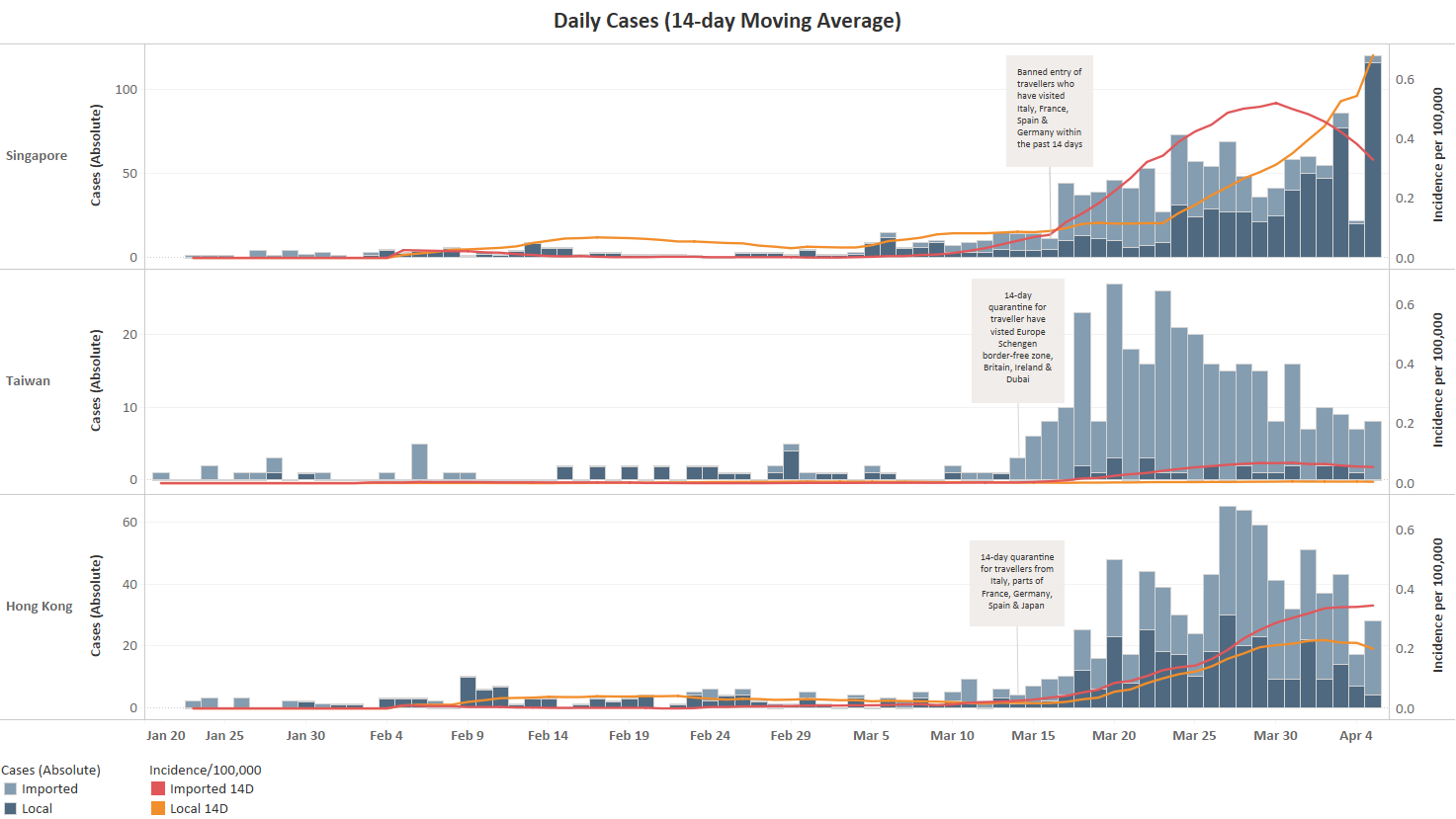


Figure S13: Daily new imported & local cases in Singapore, Taiwan & Hong Kong (14-day moving average; first Europe related restriction highlighted)

#### Imported Cases


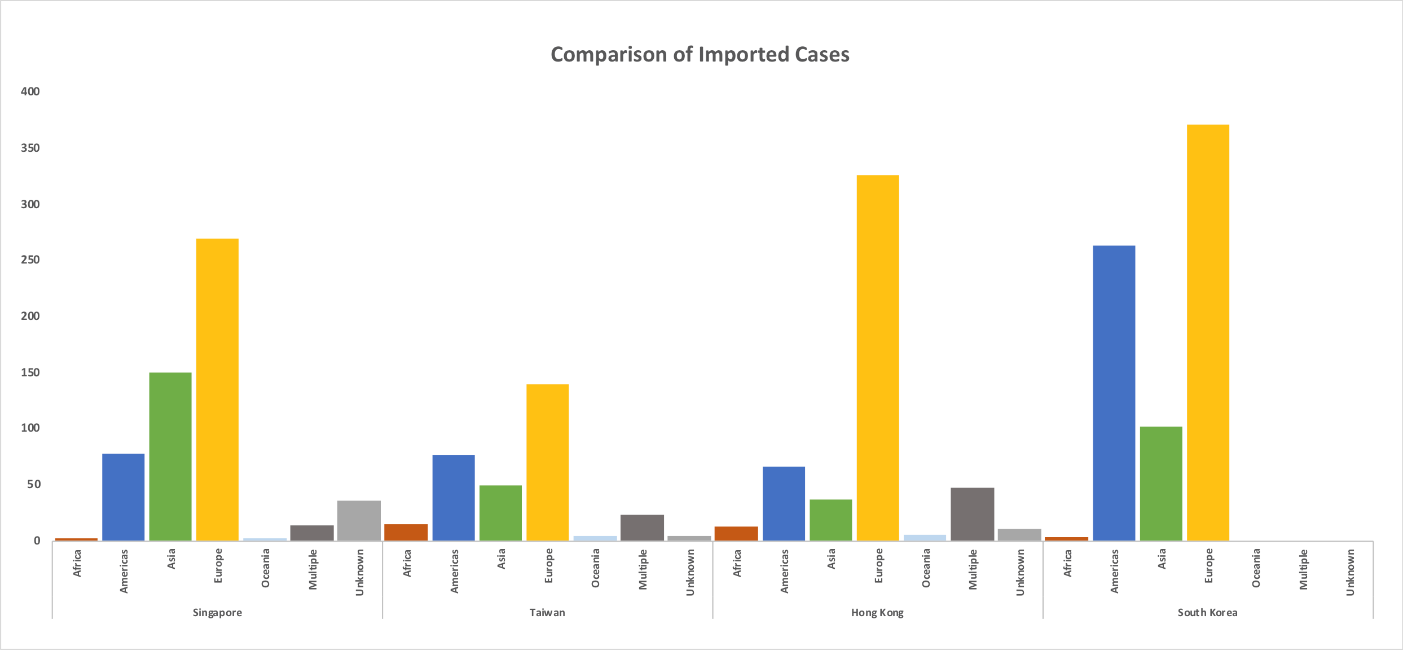


Figure S14: Source region of imported cases

#### Rate of Change (Local Cases)


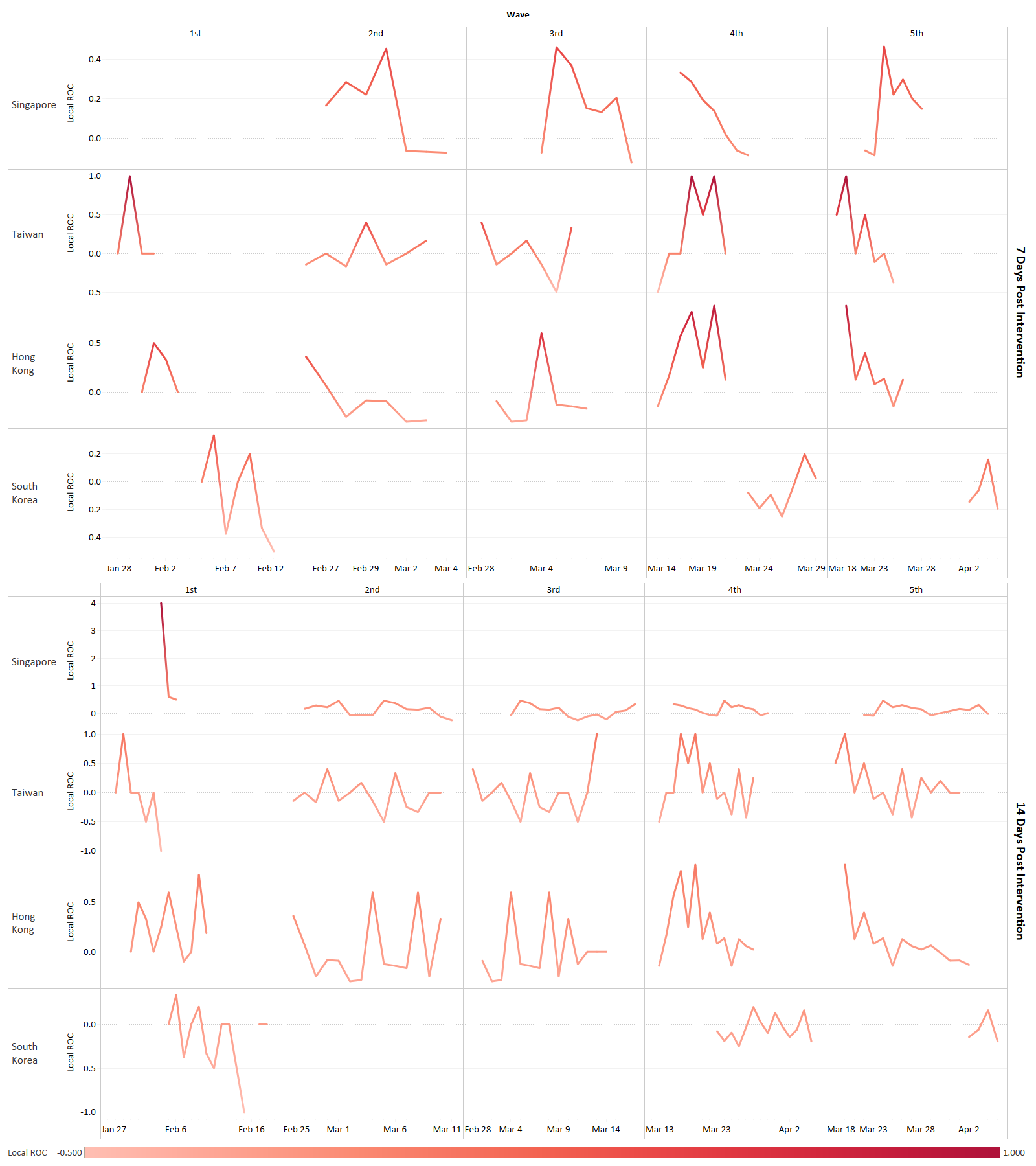


Figure S15: Rate of change in local cases following first intervention of each wave (5-day moving average; 7 & 14 days post intervention)


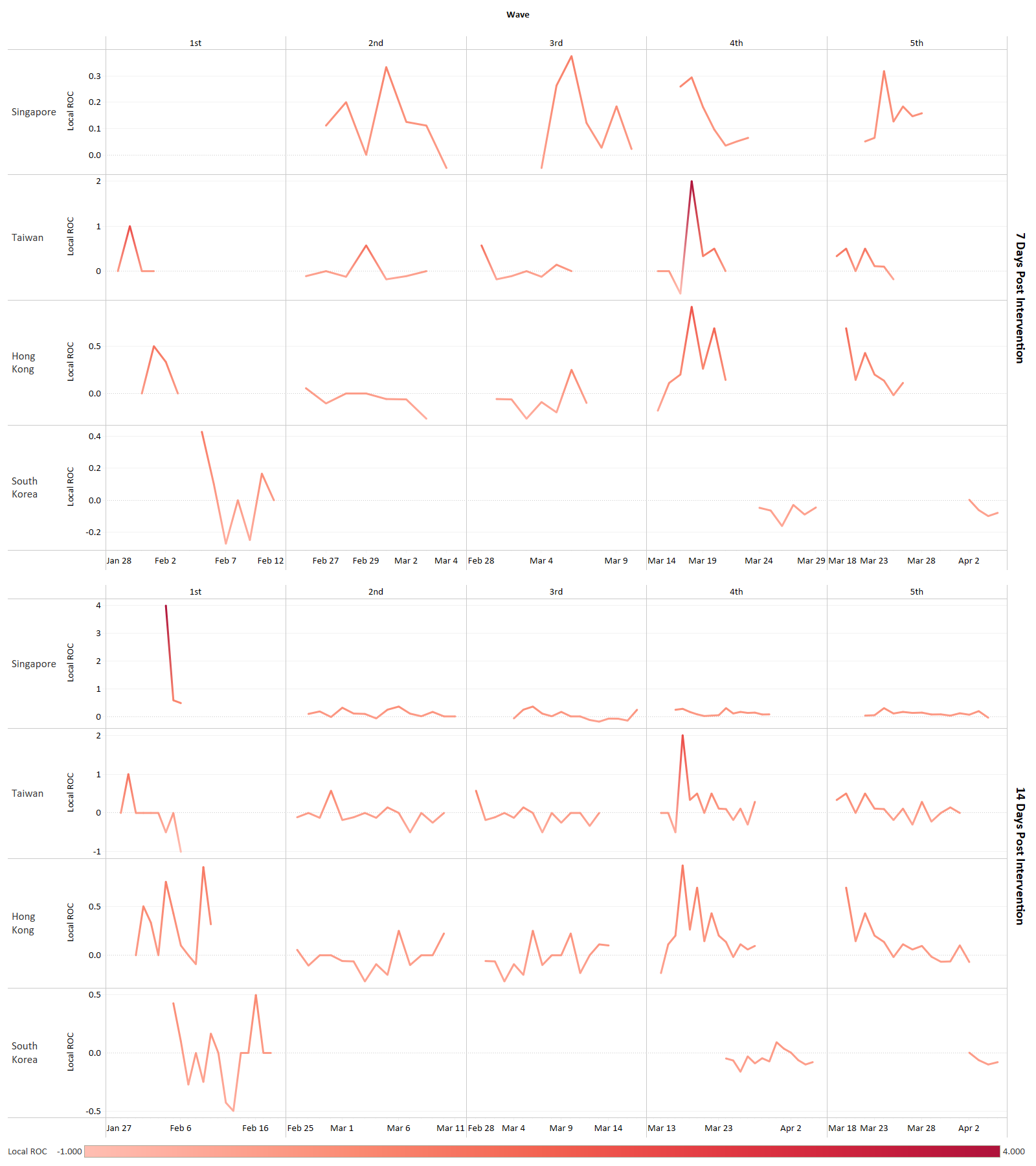


Figure S16: Rate of change in local cases following first intervention of each wave (7-day moving average; 7 & 14 days post intervention)


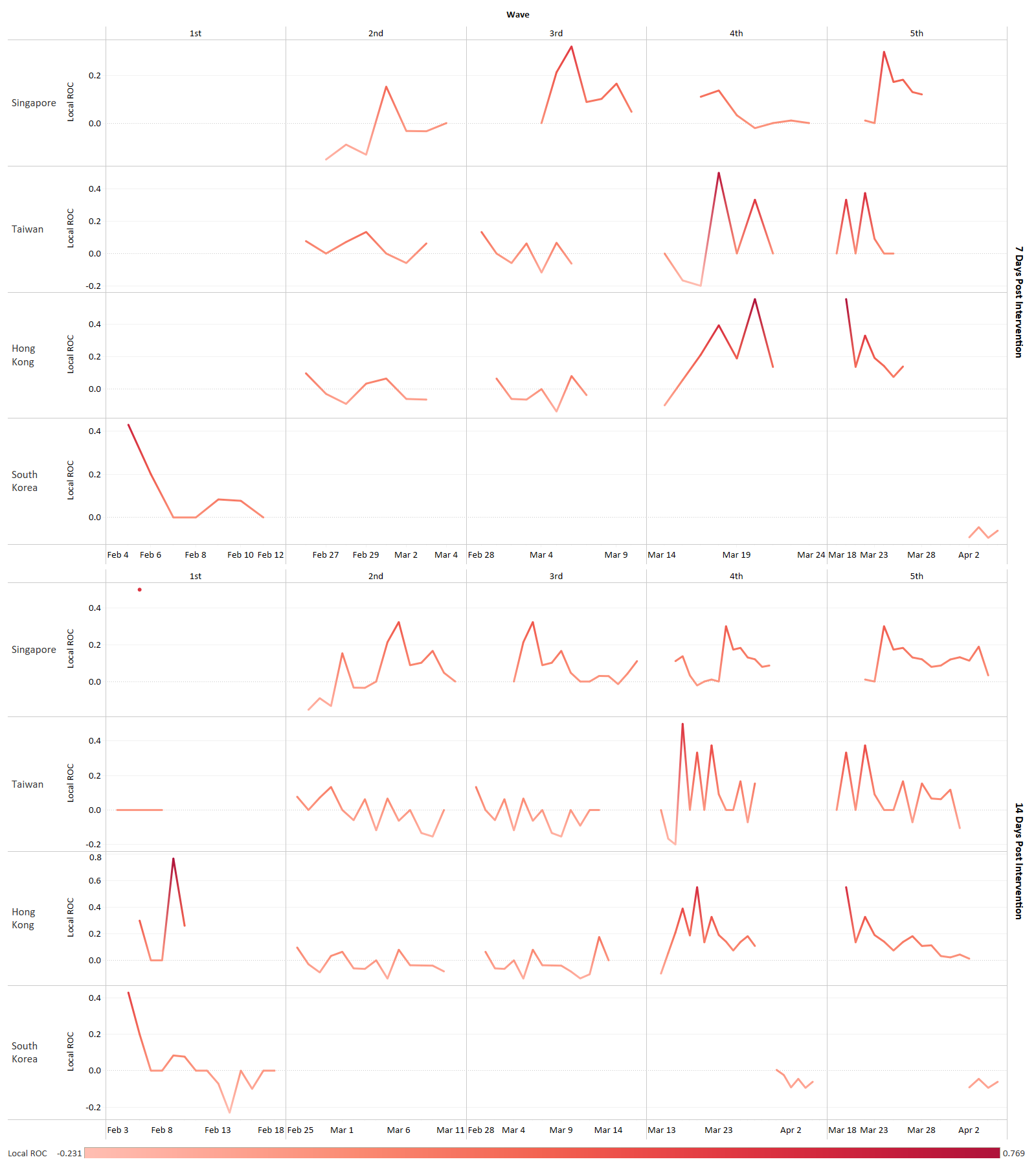


Figure S17: Rate of change in local cases following first intervention of each wave (14-day moving average; 7 & 14 days post intervention)

## II. Tables

#### Correlation of rate of change in imported and local cases after first intervention of each wave

**Using 5-Day Moving Average of Cases**

Table S1: Correlation between rate of change in imported and local cases 7 days post intervention

|  | **Singapore** | **Taiwan** | **Hong Kong** | **South Korea** |
| --- | --- | --- | --- | --- |
| Wave 1 | NA | 0.565916458 | 0.907218423 | 0.658673581 |
| Wave 2 | NA | 0.506325331 | 0.428988039 | NA |
| Wave 3 | 0.588176482 | 0.208259185 | 0.008953163 | NA |
| Wave 4 | 0.793719163 | -0.197964339 | 0.796659433 | -0.304813233 |
| Wave 5 | 0.114366887 | 0.72172779 | 0.925286716 | -0.808930066 |

Table S2: Correlation between rate of change in imported and local cases 14 days post intervention

|  | **Singapore** | **Taiwan** | **Hong Kong** | **South Korea** |
| --- | --- | --- | --- | --- |
| Wave 1 | 0.79594418 | -0.362640722 | 0.009610608 | 0.719171522 |
| Wave 2 | 0.566119281 | 0.220218163 | 0.342170157 | NA |
| Wave 3 | 0.5069856 | -0.130938249 | 0.212813434 | NA |
| Wave 4 | 0.408792483 | 0.092220707 | 0.756058094 | -0.337238702 |
| Wave 5 | 0.295456231 | 0.442943843 | 0.834299552 | -0.808930066 |

**Using 7-Day Moving Average of Cases**

Table S3: Correlation between rate of change in imported and local cases 7 days post intervention

|  | **Singapore** | **Taiwan** | **Hong Kong** | **South Korea** |
| --- | --- | --- | --- | --- |
| Wave 1 | NA | 0.333333333 | -0.058025885 | 0.168178836 |
| Wave 2 | -1 | 0.763906173 | 0.356476274 | NA |
| Wave 3 | 0.689291652 | 0.710393246 | -0.045437789 | NA |
| Wave 4 | 0.598654968 | 0.156021047 | 0.287081691 | -0.383106987 |
| Wave 5 | -0.482777259 | 0.533610104 | 0.938952223 | -0.26719254 |

Table S4: Correlation between rate of change in imported and local cases 14 days post intervention

|  | **Singapore** | **Taiwan** | **Hong Kong** | **South Korea** |
| --- | --- | --- | --- | --- |
| Wave 1 | 0.956067947 | -0.604229577 | -0.240169088 | -0.085954817 |
| Wave 2 | 0.343415565 | 0.578322398 | 0.317049989 | NA |
| Wave 3 | 0.677510579 | 0.566112934 | 0.010363368 | NA |
| Wave 4 | 0.278334894 | 0.27771245 | 0.388282713 | -0.441541796 |
| Wave 5 | 0.064354511 | 0.543540994 | 0.852189404 | -0.26719254 |

**Using 14-Day Moving Average of Cases**

Table S5: Correlation between rate of change in imported and local cases 7 days post intervention

|  | **Singapore** | **Taiwan** | **Hong Kong** | **South Korea** |
| --- | --- | --- | --- | --- |
| Wave 1 | NA | NA | NA | 0.858389788 |
| Wave 2 | -0.201901287 | 0.691156949 | 0.68843774 | NA |
| Wave 3 | -0.105940669 | 0.732188221 | 0.692048452 | NA |
| Wave 4 | 0.608676658 | 0.208935164 | 0.756050614 | NA |
| Wave 5 | -0.093420902 | 0.480665629 | 0.935284835 | 0.760346329 |

Table S6: Correlation between rate of change in imported and local cases 14 days post intervention

|  | **Singapore** | **Taiwan** | **Hong Kong** | **South Korea** |
| --- | --- | --- | --- | --- |
| Wave 1 | NA | NA | -0.844464025 | 0.786908544 |
| Wave 2 | 0.283358821 | 0.705413058 | 0.385326155 | NA |
| Wave 3 | 0.130969064 | 0.620253545 | -0.063187605 | NA |
| Wave 4 | -0.136248367 | 0.047067864 | 0.676367963 | 0.902353951 |
| Wave 5 | 0.071864223 | 0.475044372 | 0.881306215 | 0.760346329 |

#### Rate of Change

Table S7: Windows of Interest

|  | **1st (against China)** | | | **2nd (against South Korea)** | | | **3rd (against Iran or Italy)** | | | **4th (against Europe)** | | | **5th (against all countries)** | | | **Window (Days)** |
| --- | --- | --- | --- | --- | --- | --- | --- | --- | --- | --- | --- | --- | --- | --- | --- | --- |
|  | Implementation | Start | End | Implementation | Start | End | Implementation | Start | End | Implementation | Start | End | Implementation | Start | End |  |
| Singapore | 1/23 | 1/24 | 1/28 | 2/26 | 2/27 | 3/2 | 3/3 | 3/4 | 3/8 | 3/16 | 3/17 | 3/21 | 3/21 | 3/22 | 3/26 | 5 |
| Taiwan | 1/25 | 1/26 | 1/30 | 2/25 | 2/26 | 3/1 | 2/28 | 2/29 | 3/4 | 3/14 | 3/15 | 3/19 | 3/18 | 3/19 | 3/23 |  |
| Hong Kong | 1/27 | 1/28 | 2/1 | 2/25 | 2/26 | 3/1 | 2/29 | 3/1 | 3/5 | 3/14 | 3/15 | 3/19 | 3/19 | 3/20 | 3/24 |  |
| South Korea | 2/4 | 2/5 | 2/9 | NA | NA | NA | NA | NA | NA | 3/22 | 3/23 | 3/27 | 4/1 | 4/2 | 4/6 |  |
| Singapore | 1/23 | 1/24 | 1/30 | 2/26 | 2/27 | 3/4 | 3/3 | 3/4 | 3/10 | 3/16 | 3/17 | 3/23 | 3/21 | 3/22 | 3/28 | 7 |
| Taiwan | 1/25 | 1/26 | 2/1 | 2/25 | 2/26 | 3/3 | 2/28 | 2/29 | 3/6 | 3/14 | 3/15 | 3/21 | 3/18 | 3/19 | 3/25 |  |
| Hong Kong | 1/27 | 1/28 | 2/3 | 2/25 | 2/26 | 3/3 | 2/29 | 3/1 | 3/7 | 3/14 | 3/15 | 3/21 | 3/19 | 3/20 | 3/26 |  |
| South Korea | 2/4 | 2/5 | 2/11 | NA | NA | NA | NA | NA | NA | 3/22 | 3/23 | 3/29 | 4/1 | 4/2 | 4/8 |  |
| Singapore | 1/23 | 1/24 | 2/6 | 2/26 | 2/27 | 3/11 | 3/3 | 3/4 | 3/17 | 3/16 | 3/17 | 3/30 | 3/21 | 3/22 | 4/4 | 14 |
| Taiwan | 1/25 | 1/26 | 2/8 | 2/25 | 2/26 | 3/10 | 2/28 | 2/29 | 3/13 | 3/14 | 3/15 | 3/28 | 3/18 | 3/19 | 4/1 |  |
| Hong Kong | 1/27 | 1/28 | 2/10 | 2/25 | 2/26 | 3/10 | 2/29 | 3/1 | 3/14 | 3/14 | 3/15 | 3/28 | 3/19 | 3/20 | 4/2 |  |
| South Korea | 2/4 | 2/5 | 2/18 | NA | NA | NA | NA | NA | NA | 3/22 | 3/23 | 4/5 | 4/1 | 4/2 | 4/15 |  |

Table S8: Rate of change in cases 7 days post intervention (Calculated using 5-day moving average for daily cases)

| **7 Day Post Intervention Window** | | | | | | | | | | | | |
| --- | --- | --- | --- | --- | --- | --- | --- | --- | --- | --- | --- | --- |
|  | **Date** | **Singapore Imported** | **Singapore Local** | **Date** | **Taiwan Imported** | **Taiwan Local** | **Date** | **Hong Kong Imported** | **Hong Kong Local** | **Date** | **South Korea Imported** | **South Korea Local** |
| 1st Wave | 1/24 |  |  | 1/26 | 0 |  | 1/28 | -0.25 |  | 2/5 | 0.333333333 | 0 |
|  | 1/25 |  |  | 1/27 | 0.333333333 |  | 1/29 | -0.166666667 |  | 2/6 | 0 | 0.333333333 |
|  | 1/26 |  |  | 1/28 | 0.5 |  | 1/30 | 0 |  | 2/7 | 0 | -0.375 |
|  | 1/27 |  |  | 1/29 | -0.333333333 | 0 | 1/31 | -0.4 | 0 | 2/8 | 0 | 0 |
|  | 1/28 | 0 |  | 1/30 | 0 | 1 | 2/1 | 0 | 0.5 | 2/9 | 0.25 | 0.2 |
|  | 1/29 | 0.428571429 |  | 1/31 | 0 | 0 | 2/2 | 0 | 0.333333333 | 2/10 | -0.4 | -0.333333333 |
|  | 1/30 | 0.1 |  | 2/1 | -0.25 | 0 | 2/3 | -0.666666667 | 0 | 2/11 | -0.333333333 | -0.5 |
|  | **Date** | **Singapore Imported** | **Singapore Local** | **Date** | **Taiwan Imported** | **Taiwan Local** | **Date** | **Hong Kong Imported** | **Hong Kong Local** | **Date** | **South Korea Imported** | **South Korea Local** |
| 2nd Wave | 2/27 |  | 0.166666667 | 2/26 |  | -0.142857143 | 2/26 | 0.4 | 0.363636364 |  |  |  |
|  | 2/28 |  | 0.285714286 | 2/27 |  | 0 | 2/27 | 0 | 0.066666667 |  |  |  |
|  | 2/29 |  | 0.222222222 | 2/28 |  | -0.166666667 | 2/28 | 0 | -0.25 |  |  |  |
|  | 3/1 |  | 0.454545455 | 2/29 | 1 | 0.4 | 2/29 | -0.571428571 | -0.083333333 |  |  |  |
|  | 3/2 |  | -0.0625 | 3/1 | 0.5 | -0.142857143 | 3/1 | 0.666666667 | -0.090909091 |  |  |  |
|  | 3/3 |  | -0.066666667 | 3/2 | 0 | -1.70516E-16 | 3/2 | -0.4 | -0.3 |  |  |  |
|  | 3/4 |  | -0.071428571 | 3/3 | 0 | 0.166666667 | 3/3 | 0 | -0.285714286 |  |  |  |
|  | **Date** | **Singapore Imported** | **Singapore Local** | **Date** | **Taiwan Imported** | **Taiwan Local** | **Date** | **Hong Kong Imported** | **Hong Kong Local** | **Date** | **South Korea Imported** | **South Korea Local** |
| 3rd Wave | 3/4 |  | -0.071428571 | 2/29 | 1 | 0.4 | 3/1 | 0.666666667 | -0.090909091 |  |  |  |
|  | 3/5 | 1 | 0.461538462 | 3/1 | 0.5 | -0.142857143 | 3/2 | -0.4 | -0.3 |  |  |  |
|  | 3/6 | 1.5 | 0.368421053 | 3/2 | 0 | -1.70516E-16 | 3/3 | 0 | -0.285714286 |  |  |  |
|  | 3/7 | 0 | 0.153846154 | 3/3 | 0 | 0.166666667 | 3/4 | 0 | 0.6 |  |  |  |
|  | 3/8 | 0.6 | 0.133333333 | 3/4 | -0.333333333 | -0.142857143 | 3/5 | 0 | -0.125 |  |  |  |
|  | 3/9 | 0 | 0.205882353 | 3/5 | 0 | -0.5 | 3/6 | -0.333333333 | -0.142857143 |  |  |  |
|  | 3/10 | 0.375 | -0.12195122 | 3/6 | -0.5 | 0.333333333 | 3/7 | 1 | -0.166666667 |  |  |  |
|  | **Date** | **Singapore Imported** | **Singapore Local** | **Date** | **Taiwan Imported** | **Taiwan Local** | **Date** | **Hong Kong Imported** | **Hong Kong Local** | **Date** | **South Korea Imported** | **South Korea Local** |
| 4th Wave | 3/17 | 0.627906977 | 0.333333333 | 3/15 | 0.833333333 | -0.5 | 3/15 | 0.157894737 | -0.142857143 | 3/23 | 0.161764706 | -0.078585462 |
|  | 3/18 | 0.2 | 0.285714286 | 3/16 | 0.636363636 | 0 | 3/16 | -0.045454545 | 0.166666667 | 3/24 | 0.164556962 | -0.189765458 |
|  | 3/19 | 0.214285714 | 0.194444444 | 3/17 | 0.5 | 0 | 3/17 | 0.19047619 | 0.571428571 | 3/25 | 0.532608696 | -0.094736842 |
|  | 3/20 | 0.254901961 | 0.139534884 | 3/18 | 0.777777778 | 1 | 3/18 | 0.4 | 0.818181818 | 3/26 | 0.304964539 | -0.25 |
|  | 3/21 | 0.2265625 | 0.020408163 | 3/19 | 0.083333333 | 0.5 | 3/19 | 0.2 | 0.25 | 3/27 | 0.010869565 | -0.034883721 |
|  | 3/22 | 0.076433121 | -0.06 | 3/20 | 0.346153846 | 1 | 3/20 | 0.452380952 | 0.88 | 3/28 | 0.177419355 | 0.196787149 |
|  | 3/23 | -0.035502959 | -0.085106383 | 3/21 | 0.142857143 | 0 | 3/21 | 0.032786885 | 0.127659574 | 3/29 | 0.100456621 | 0.023489933 |
|  | **Date** | **Singapore Imported** | **Singapore Local** | **Date** | **Taiwan Imported** | **Taiwan Local** | **Date** | **Hong Kong Imported** | **Hong Kong Local** | **Date** | **South Korea Imported** | **South Korea Local** |
| 5th Wave | 3/22 | 0.076433121 | -0.06 | 3/19 | 0.083333333 | 0.5 | 3/20 | 0.452380952 | 0.88 | 4/2 | -0.051792829 | -0.144736842 |
|  | 3/23 | -0.035502959 | -0.085106383 | 3/20 | 0.346153846 | 1 | 3/21 | 0.032786885 | 0.127659574 | 4/3 | -0.012605042 | -0.061538462 |
|  | 3/24 | 0.085889571 | 0.465116279 | 3/21 | 0.142857143 | 0 | 3/22 | 0.206349206 | 0.396226415 | 4/4 | -0.09787234 | 0.159836066 |
|  | 3/25 | -0.016949153 | 0.222222222 | 3/22 | 0.0375 | 0.5 | 3/23 | 0.105263158 | 0.081081081 | 4/5 | 0.051886792 | -0.19434629 |
|  | 3/26 | -0.057471264 | 0.298701299 | 3/23 | 0.048192771 | -0.111111111 | 3/24 | 0.035714286 | 0.1375 | 4/6 |  |  |
|  | 3/27 | -0.024390244 | 0.2 | 3/24 | 0.149425287 | -2.55774E-16 | 3/25 | -0.126436782 | -0.142857143 | 4/7 |  |  |
|  | 3/28 | 0.01875 | 0.15 | 3/25 | -0.04 | -0.375 | 3/26 | 0.210526316 | 0.128205128 | 4/8 |  |  |

Table S9: Rate of change in cases 14 days post intervention (Calculated using 5-day moving average for daily cases)

| **14 Day Post Intervention Window** | | | | | | | | | | | | |
| --- | --- | --- | --- | --- | --- | --- | --- | --- | --- | --- | --- | --- |
|  | **Date** | **Singapore Imported** | **Singapore Local** | **Date** | **Taiwan Imported** | **Taiwan Local** | **Date** | **Hong Kong Imported** | **Hong Kong Local** | **Date** | **South Korea Imported** | **South Korea Local** |
| 1st Wave | 1/24 |  |  | 1/26 | 0 |  | 1/28 | -0.25 |  | 2/5 | 0.333333333 | 0 |
|  | 1/25 |  |  | 1/27 | 0.333333333 |  | 1/29 | -0.166666667 |  | 2/6 | 0 | 0.333333333 |
|  | 1/26 |  |  | 1/28 | 0.5 |  | 1/30 | 0 |  | 2/7 | 0 | -0.375 |
|  | 1/27 |  |  | 1/29 | -0.333333333 | 0 | 1/31 | -0.4 | 0 | 2/8 | 0 | 0 |
|  | 1/28 | 0 |  | 1/30 | 0 | 1 | 2/1 | 0 | 0.5 | 2/9 | 0.25 | 0.2 |
|  | 1/29 | 0.428571429 |  | 1/31 | 0 | 0 | 2/2 | 0 | 0.333333333 | 2/10 | -0.4 | -0.333333333 |
|  | 1/30 | 0.1 |  | 2/1 | -0.25 | 0 | 2/3 | -0.666666667 | 0 | 2/11 | -0.333333333 | -0.5 |
|  | 1/31 | 0.272727273 |  | 2/2 | -0.666666667 | -0.5 | 2/4 | 0 | 0.25 | 2/12 | 0 | 0 |
|  | 2/1 | -0.214285714 |  | 2/3 | 0 | 0 | 2/5 | -1 | 0.6 | 2/13 | 0 | 0 |
|  | 2/2 | -0.090909091 |  | 2/4 | 1 | -1 | 2/6 |  | 0.25 | 2/14 | -1 | -0.5 |
|  | 2/3 | -0.2 |  | 2/5 | -0.5 |  | 2/7 |  | -0.1 | 2/15 |  | -1 |
|  | 2/4 | -0.125 | 4 | 2/6 | 5 |  | 2/8 | 0 | 0 | 2/16 |  |  |
|  | 2/5 | -0.428571429 | 0.6 | 2/7 | 0 |  | 2/9 | 0 | 0.777777778 | 2/17 | 0 | 0 |
|  | 2/6 | -0.25 | 0.5 | 2/8 | 0.166666667 |  | 2/10 | 0 | 0.1875 | 2/18 | 0 | 0 |
|  | **Date** | **Singapore Imported** | **Singapore Local** | **Date** | **Taiwan Imported** | **Taiwan Local** | **Date** | **Hong Kong Imported** | **Hong Kong Local** | **Date** | **South Korea Imported** | **South Korea Local** |
| 2nd Wave | 2/27 |  | 0.166666667 | 2/26 |  | -0.142857143 | 2/26 | 0.4 | 0.363636364 |  |  |  |
|  | 2/28 |  | 0.285714286 | 2/27 |  | 0 | 2/27 | 0 | 0.066666667 |  |  |  |
|  | 2/29 |  | 0.222222222 | 2/28 |  | -0.166666667 | 2/28 | 0 | -0.25 |  |  |  |
|  | 3/1 |  | 0.454545455 | 2/29 | 1 | 0.4 | 2/29 | -0.571428571 | -0.083333333 |  |  |  |
|  | 3/2 |  | -0.0625 | 3/1 | 0.5 | -0.142857143 | 3/1 | 0.666666667 | -0.090909091 |  |  |  |
|  | 3/3 |  | -0.066666667 | 3/2 | 0 | -1.70516E-16 | 3/2 | -0.4 | -0.3 |  |  |  |
|  | 3/4 |  | -0.071428571 | 3/3 | 0 | 0.166666667 | 3/3 | 0 | -0.285714286 |  |  |  |
|  | 3/5 | 1 | 0.461538462 | 3/4 | -0.333333333 | -0.142857143 | 3/4 | 0 | 0.6 |  |  |  |
|  | 3/6 | 1.5 | 0.368421053 | 3/5 | 0 | -0.5 | 3/5 | 0 | -0.125 |  |  |  |
|  | 3/7 | 0 | 0.153846154 | 3/6 | -0.5 | 0.333333333 | 3/6 | -0.333333333 | -0.142857143 |  |  |  |
|  | 3/8 | 0.6 | 0.133333333 | 3/7 | 0 | -0.25 | 3/7 | 1 | -0.166666667 |  |  |  |
|  | 3/9 | 0 | 0.205882353 | 3/8 | 0 | -0.333333333 | 3/8 | 0.5 | 0.6 |  |  |  |
|  | 3/10 | 0.375 | -0.12195122 | 3/9 | 0 | 0 | 3/9 | -0.166666667 | -0.25 |  |  |  |
|  | 3/11 | 0.272727273 | -0.25 | 3/10 | 0 | 0 | 3/10 | 0.6 | 0.333333333 |  |  |  |
|  | **Date** | **Singapore Imported** | **Singapore Local** | **Date** | **Taiwan Imported** | **Taiwan Local** | **Date** | **Hong Kong Imported** | **Hong Kong Local** | **Date** | **South Korea Imported** | **South Korea Local** |
| 3rd Wave | 3/4 |  | -0.071428571 | 2/29 | 1 | 0.4 | 3/1 | 0.666666667 | -0.090909091 |  |  |  |
|  | 3/5 | 1 | 0.461538462 | 3/1 | 0.5 | -0.142857143 | 3/2 | -0.4 | -0.3 |  |  |  |
|  | 3/6 | 1.5 | 0.368421053 | 3/2 | 0 | -1.70516E-16 | 3/3 | 0 | -0.285714286 |  |  |  |
|  | 3/7 | 0 | 0.153846154 | 3/3 | 0 | 0.166666667 | 3/4 | 0 | 0.6 |  |  |  |
|  | 3/8 | 0.6 | 0.133333333 | 3/4 | -0.333333333 | -0.142857143 | 3/5 | 0 | -0.125 |  |  |  |
|  | 3/9 | 0 | 0.205882353 | 3/5 | 0 | -0.5 | 3/6 | -0.333333333 | -0.142857143 |  |  |  |
|  | 3/10 | 0.375 | -0.12195122 | 3/6 | -0.5 | 0.333333333 | 3/7 | 1 | -0.166666667 |  |  |  |
|  | 3/11 | 0.272727273 | -0.25 | 3/7 | 0 | -0.25 | 3/8 | 0.5 | 0.6 |  |  |  |
|  | 3/12 | 0.5 | -0.111111111 | 3/8 | 0 | -0.333333333 | 3/9 | -0.166666667 | -0.25 |  |  |  |
|  | 3/13 | 0.333333333 | -0.041666667 | 3/9 | 0 | 0 | 3/10 | 0.6 | 0.333333333 |  |  |  |
|  | 3/14 | 0.321428571 | -0.217391304 | 3/10 | 0 | 0 | 3/11 | 0.875 | -0.125 |  |  |  |
|  | 3/15 | 0.162162162 | 0.055555556 | 3/11 | 1 | -0.5 | 3/12 | 0 | 0 |  |  |  |
|  | 3/16 | -1.84078E-16 | 0.105263158 | 3/12 | 0.5 | 0 | 3/13 | 0.066666667 | 0 |  |  |  |
|  | 3/17 | 0.627906977 | 0.333333333 | 3/13 | 0 | 1 | 3/14 | 0.1875 | 0 |  |  |  |
|  | **Date** | **Singapore Imported** | **Singapore Local** | **Date** | **Taiwan Imported** | **Taiwan Local** | **Date** | **Hong Kong Imported** | **Hong Kong Local** | **Date** | **South Korea Imported** | **South Korea Local** |
| 4th Wave | 3/17 | 0.627906977 | 0.333333333 | 3/15 | 0.833333333 | -0.5 | 3/15 | 0.157894737 | -0.142857143 | 3/23 | 0.161764706 | -0.078585462 |
|  | 3/18 | 0.2 | 0.285714286 | 3/16 | 0.636363636 | 0 | 3/16 | -0.045454545 | 0.166666667 | 3/24 | 0.164556962 | -0.189765458 |
|  | 3/19 | 0.214285714 | 0.194444444 | 3/17 | 0.5 | 0 | 3/17 | 0.19047619 | 0.571428571 | 3/25 | 0.532608696 | -0.094736842 |
|  | 3/20 | 0.254901961 | 0.139534884 | 3/18 | 0.777777778 | 1 | 3/18 | 0.4 | 0.818181818 | 3/26 | 0.304964539 | -0.25 |
|  | 3/21 | 0.2265625 | 0.020408163 | 3/19 | 0.083333333 | 0.5 | 3/19 | 0.2 | 0.25 | 3/27 | 0.010869565 | -0.034883721 |
|  | 3/22 | 0.076433121 | -0.06 | 3/20 | 0.346153846 | 1 | 3/20 | 0.452380952 | 0.88 | 3/28 | 0.177419355 | 0.196787149 |
|  | 3/23 | -0.035502959 | -0.085106383 | 3/21 | 0.142857143 | 0 | 3/21 | 0.032786885 | 0.127659574 | 3/29 | 0.100456621 | 0.023489933 |
|  | 3/24 | 0.085889571 | 0.465116279 | 3/22 | 0.0375 | 0.5 | 3/22 | 0.206349206 | 0.396226415 | 3/30 | 0.033195021 | -0.098360656 |
|  | 3/25 | -0.016949153 | 0.222222222 | 3/23 | 0.048192771 | -0.111111111 | 3/23 | 0.105263158 | 0.081081081 | 3/31 | -0.060240964 | 0.130909091 |
|  | 3/26 | -0.057471264 | 0.298701299 | 3/24 | 0.149425287 | -2.55774E-16 | 3/24 | 0.035714286 | 0.1375 | 4/1 | 0.072649573 | -0.022508039 |
|  | 3/27 | -0.024390244 | 0.2 | 3/25 | -0.04 | -0.375 | 3/25 | -0.126436782 | -0.142857143 | 4/2 | -0.051792829 | -0.144736842 |
|  | 3/28 | 0.01875 | 0.15 | 3/26 | -0.041666667 | 0.4 | 3/26 | 0.210526316 | 0.128205128 | 4/3 | -0.012605042 | -0.061538462 |
|  | 3/29 | -0.165644172 | -0.072463768 | 3/27 | 0.02173913 | -0.428571429 | 3/27 | 0.173913043 | 0.056818182 | 4/4 | -0.09787234 | 0.159836066 |
|  | 3/30 | -0.125 | 0.0078125 | 3/28 | -0.117021277 | 0.25 | 3/28 | 0.212962963 | 0.021505376 | 4/5 | 0.051886792 | -0.19434629 |
|  | **Date** | **Singapore Imported** | **Singapore Local** | **Date** | **Taiwan Imported** | **Taiwan Local** | **Date** | **Hong Kong Imported** | **Hong Kong Local** | **Date** | **South Korea Imported** | **South Korea Local** |
| 5th Wave | 3/22 | 0.076433121 | -0.06 | 3/19 | 0.083333333 | 0.5 | 3/20 | 0.452380952 | 0.88 | 4/2 | -0.051792829 | -0.144736842 |
|  | 3/23 | -0.035502959 | -0.085106383 | 3/20 | 0.346153846 | 1 | 3/21 | 0.032786885 | 0.127659574 | 4/3 | -0.012605042 | -0.061538462 |
|  | 3/24 | 0.085889571 | 0.465116279 | 3/21 | 0.142857143 | 0 | 3/22 | 0.206349206 | 0.396226415 | 4/4 | -0.09787234 | 0.159836066 |
|  | 3/25 | -0.016949153 | 0.222222222 | 3/22 | 0.0375 | 0.5 | 3/23 | 0.105263158 | 0.081081081 | 4/5 | 0.051886792 | -0.19434629 |
|  | 3/26 | -0.057471264 | 0.298701299 | 3/23 | 0.048192771 | -0.111111111 | 3/24 | 0.035714286 | 0.1375 | 4/6 |  |  |
|  | 3/27 | -0.024390244 | 0.2 | 3/24 | 0.149425287 | -2.55774E-16 | 3/25 | -0.126436782 | -0.142857143 | 4/7 |  |  |
|  | 3/28 | 0.01875 | 0.15 | 3/25 | -0.04 | -0.375 | 3/26 | 0.210526316 | 0.128205128 | 4/8 |  |  |
|  | 3/29 | -0.165644172 | -0.072463768 | 3/26 | -0.041666667 | 0.4 | 3/27 | 0.173913043 | 0.056818182 | 4/9 |  |  |
|  | 3/30 | -0.125 | 0.0078125 | 3/27 | 0.02173913 | -0.428571429 | 3/28 | 0.212962963 | 0.021505376 | 4/10 |  |  |
|  | 3/31 | -0.058823529 | 0.085271318 | 3/28 | -0.117021277 | 0.25 | 3/29 | 0.175572519 | 0.063157895 | 4/11 |  |  |
|  | 4/1 | -0.285714286 | 0.164285714 | 3/29 | -0.072289157 | 0 | 3/30 | 0.116883117 | -0.00990099 | 4/12 |  |  |
|  | 4/2 | -0.1625 | 0.122699387 | 3/30 | -0.168831169 | 0.2 | 3/31 | -0.011627907 | -0.09 | 4/13 |  |  |
|  | 4/3 | -0.089552239 | 0.306010929 | 3/31 | 0 | 1.70516E-16 | 4/1 | -0.035294118 | -0.087912088 | 4/14 |  |  |
|  | 4/4 | -0.229508197 | -0.020920502 | 4/1 | -0.125 | 0 | 4/2 | -0.097560976 | -0.13253012 | 4/15 |  |  |

Table S10: Rate of change in cases 7 days post intervention (Calculated using 7-day moving average for daily cases)

| **7 Days Post Intervention** | | | | | | | | | | | | |
| --- | --- | --- | --- | --- | --- | --- | --- | --- | --- | --- | --- | --- |
|  | **Date** | **Singapore Imported** | **Singapore Local** | **Date** | **Taiwan Imported** | **Taiwan Local** | **Date** | **Hong Kong Imported** | **Hong Kong Local** | **Date** | **South Korea Imported** | **South Korea Local** |
| 1st Wave | 1/24 |  |  | 1/26 |  |  | 1/28 |  |  | 2/5 | 0.4 | 0.428571429 |
|  | 1/25 |  |  | 1/27 |  |  | 1/29 |  |  | 2/6 | -0.142857143 | 0.1 |
|  | 1/26 |  |  | 1/28 | 0.2 |  | 1/30 | -0.2 |  | 2/7 | -0.166666667 | -0.272727273 |
|  | 1/27 |  |  | 1/29 | 0 | 0 | 1/31 | -0.25 | 0 | 2/8 | -0.2 | 0 |
|  | 1/28 |  |  | 1/30 | 0 | 1 | 2/1 | 0 | 0.5 | 2/9 | 0.5 | -0.25 |
|  | 1/29 |  |  | 1/31 | -0.166666667 | 0 | 2/2 | -0.5 | 0.333333333 | 2/10 | 0 | 0.166666667 |
|  | 1/30 | 0.083333333 |  | 2/1 | 0 | 0 | 2/3 | 0 | 0 | 2/11 | -0.166666667 | 0 |
|  | **Date** | **Singapore Imported** | **Singapore Local** | **Date** | **Taiwan Imported** | **Taiwan Local** | **Date** | **Hong Kong Imported** | **Hong Kong Local** | **Date** | **South Korea Imported** | **South Korea Local** |
| 2nd Wave | 2/27 | 0 | 0.111111111 | 2/26 |  | -0.111111111 | 2/26 | 0.4 | 0.055555556 |  |  |  |
|  | 2/28 | -1 | 0.2 | 2/27 |  | 0 | 2/27 | 0 | -0.105263158 |  |  |  |
|  | 2/29 |  | 0 | 2/28 |  | -0.125 | 2/28 | 0.142857143 | 0 |  |  |  |
|  | 3/1 |  | 0.333333333 | 2/29 | 1 | 0.571428571 | 2/29 | 0 | 0 |  |  |  |
|  | 3/2 |  | 0.125 | 3/1 | 0.5 | -0.181818182 | 3/1 | 0.125 | -0.058823529 |  |  |  |
|  | 3/3 |  | 0.111111111 | 3/2 | 0 | -0.111111111 | 3/2 | -0.444444444 | -0.0625 |  |  |  |
|  | 3/4 |  | -0.05 | 3/3 | 0 | 0 | 3/3 | 0 | -0.266666667 |  |  |  |
|  | **Date** | **Singapore Imported** | **Singapore Local** | **Date** | **Taiwan Imported** | **Taiwan Local** | **Date** | **Hong Kong Imported** | **Hong Kong Local** | **Date** | **South Korea Imported** | **South Korea Local** |
| 3rd Wave | 3/4 |  | -0.05 | 2/29 | 1 | 0.571428571 | 3/1 | 0.125 | -0.058823529 |  |  |  |
|  | 3/5 | 1 | 0.263157895 | 3/1 | 0.5 | -0.181818182 | 3/2 | -0.444444444 | -0.0625 |  |  |  |
|  | 3/6 | 1.5 | 0.375 | 3/2 | 0 | -0.111111111 | 3/3 | 0 | -0.266666667 |  |  |  |
|  | 3/7 | 0 | 0.121212121 | 3/3 | 0 | 0 | 3/4 | -0.2 | -0.090909091 |  |  |  |
|  | 3/8 | 0.6 | 0.027027027 | 3/4 | 0 | -0.125 | 3/5 | 0 | -0.2 |  |  |  |
|  | 3/9 | 0.125 | 0.184210526 | 3/5 | 0.333333333 | 0.142857143 | 3/6 | 0 | 0.25 |  |  |  |
|  | 3/10 | 0.444444444 | 0.022222222 | 3/6 | -0.25 | 0 | 3/7 | 0.5 | -0.1 |  |  |  |
|  | **Date** | **Singapore Imported** | **Singapore Local** | **Date** | **Taiwan Imported** | **Taiwan Local** | **Date** | **Hong Kong Imported** | **Hong Kong Local** | **Date** | **South Korea Imported** | **South Korea Local** |
| 4th Wave | 3/17 | 0.566037736 | 0.259259259 | 3/15 | 1 | 0 | 3/15 | 0.19047619 | -0.181818182 | 3/23 |  |  |
|  | 3/18 | 0.21686747 | 0.294117647 | 3/16 | 0.666666667 | 0 | 3/16 | 0.28 | 0.111111111 | 3/24 | 0.234042553 | -0.047543582 |
|  | 3/19 | 0.207920792 | 0.181818182 | 3/17 | 0.45 | -0.5 | 3/17 | 0.09375 | 0.2 | 3/25 | 0.396551724 | -0.064891847 |
|  | 3/20 | 0.213114754 | 0.096153846 | 3/18 | 0.689655172 | 2 | 3/18 | 0.142857143 | 0.916666667 | 3/26 | 0.265432099 | -0.161921708 |
|  | 3/21 | 0.168918919 | 0.035087719 | 3/19 | 0.12244898 | 0.333333333 | 3/19 | 0.2 | 0.260869565 | 3/27 | 0.087804878 | -0.029723992 |
|  | 3/22 | 0.208092486 | 0.050847458 | 3/20 | 0.436363636 | 0.5 | 3/20 | 0.458333333 | 0.689655172 | 3/28 | 0.179372197 | -0.089715536 |
|  | 3/23 | 0.057416268 | 0.064516129 | 3/21 | 0.189873418 | 0 | 3/21 | 0.085714286 | 0.142857143 | 3/29 | 0.098859316 | -0.045673077 |
|  | **Date** | **Singapore Imported** | **Singapore Local** | **Date** | **Taiwan Imported** | **Taiwan Local** | **Date** | **Hong Kong Imported** | **Hong Kong Local** | **Date** | **South Korea Imported** | **South Korea Local** |
| 5th Wave | 3/22 | 0.208092486 | 0.050847458 | 3/19 | 0.12244898 | 0.333333333 | 3/20 | 0.458333333 | 0.689655172 | 4/2 | -0.048048048 | 0.002398082 |
|  | 3/23 | 0.057416268 | 0.064516129 | 3/20 | 0.436363636 | 0.5 | 3/21 | 0.085714286 | 0.142857143 | 4/3 | 0.066246057 | -0.062200957 |
|  | 3/24 | 0.036199095 | 0.318181818 | 3/21 | 0.189873418 | 0 | 3/22 | 0.171052632 | 0.428571429 | 4/4 | -0.038461538 | -0.099489796 |
|  | 3/25 | 0.03930131 | 0.126436782 | 3/22 | 0.074468085 | 0.5 | 3/23 | 0.157303371 | 0.2 | 4/5 | 0.012307692 | -0.079320113 |
|  | 3/26 | -0.012605042 | 0.183673469 | 3/23 | 0.168316832 | 0.111111111 | 3/24 | 0.067961165 | 0.135416667 | 4/6 |  |  |
|  | 3/27 | 0.025531915 | 0.146551724 | 3/24 | 0.084745763 | 0.1 | 3/25 | 0.009090909 | -0.018348624 | 4/7 |  |  |
|  | 3/28 | -0.058091286 | 0.157894737 | 3/25 | -0.0078125 | -0.181818182 | 3/26 | 0.135135135 | 0.112149533 | 4/8 |  |  |

Table S11: Rate of change in cases 14 days post intervention (Calculated using 7-day moving average for daily cases)

| **14 Days Post Intervention** | | | | | | | | | | | | |
| --- | --- | --- | --- | --- | --- | --- | --- | --- | --- | --- | --- | --- |
|  | **Date** | **Singapore Imported** | **Singapore Local** | **Date** | **Taiwan Imported** | **Taiwan Local** | **Date** | **Hong Kong Imported** | **Hong Kong Local** | **Date** | **South Korea Imported** | **South Korea Local** |
| 1st Wave | 1/24 |  |  | 1/26 |  |  | 1/28 |  |  | 2/5 | 0.4 | 0.428571429 |
|  | 1/25 |  |  | 1/27 |  |  | 1/29 |  |  | 2/6 | -0.142857143 | 0.1 |
|  | 1/26 |  |  | 1/28 | 0.2 |  | 1/30 | -0.2 |  | 2/7 | -0.166666667 | -0.272727273 |
|  | 1/27 |  |  | 1/29 | 0 | 0 | 1/31 | -0.25 | 0 | 2/8 | -0.2 | 0 |
|  | 1/28 |  |  | 1/30 | 0 | 1 | 2/1 | 0 | 0.5 | 2/9 | 0.5 | -0.25 |
|  | 1/29 |  |  | 1/31 | -0.166666667 | 0 | 2/2 | -0.5 | 0.333333333 | 2/10 | 0 | 0.166666667 |
|  | 1/30 | 0.083333333 |  | 2/1 | 0 | 0 | 2/3 | 0 | 0 | 2/11 | -0.166666667 | 0 |
|  | 1/31 | 0.153846154 |  | 2/2 | -0.2 | 0 | 2/4 | 0 | 0.75 | 2/12 | -0.4 | -0.428571429 |
|  | 2/1 | 0 |  | 2/3 | -0.25 | 0 | 2/5 | -0.666666667 | 0.428571429 | 2/13 | -0.333333333 | -0.5 |
|  | 2/2 | 0 |  | 2/4 | -0.333333333 | -0.5 | 2/6 | 0 | 0.1 | 2/14 | 0 | 0 |
|  | 2/3 | -0.133333333 |  | 2/5 | 0 | 0 | 2/7 | 1 | 0 | 2/15 | 0 | 0 |
|  | 2/4 | 0 | 4 | 2/6 | 2.5 | -1 | 2/8 | 0 | -0.090909091 | 2/16 | -1 | 0.5 |
|  | 2/5 | -0.307692308 | 0.6 | 2/7 | -0.142857143 |  | 2/9 | 0 | 0.9 | 2/17 | 0 | 0 |
|  | 2/6 | -0.222222222 | 0.5 | 2/8 | 0.166666667 |  | 2/10 | 0 | 0.315789474 | 2/18 | 0 | 0 |
|  | **Date** | **Singapore Imported** | **Singapore Local** | **Date** | **Taiwan Imported** | **Taiwan Local** | **Date** | **Hong Kong Imported** | **Hong Kong Local** | **Date** | **South Korea Imported** | **South Korea Local** |
| 2nd Wave | 2/27 | 0 | 0.111111111 | 2/26 |  | -0.111111111 | 2/26 | 0.4 | 0.055555556 |  |  |  |
|  | 2/28 | -1 | 0.2 | 2/27 |  | 0 | 2/27 | 0 | -0.105263158 |  |  |  |
|  | 2/29 |  | 0 | 2/28 |  | -0.125 | 2/28 | 0.142857143 | 0 |  |  |  |
|  | 3/1 |  | 0.333333333 | 2/29 | 1 | 0.571428571 | 2/29 | 0 | 0 |  |  |  |
|  | 3/2 |  | 0.125 | 3/1 | 0.5 | -0.181818182 | 3/1 | 0.125 | -0.058823529 |  |  |  |
|  | 3/3 |  | 0.111111111 | 3/2 | 0 | -0.111111111 | 3/2 | -0.444444444 | -0.0625 |  |  |  |
|  | 3/4 |  | -0.05 | 3/3 | 0 | 0 | 3/3 | 0 | -0.266666667 |  |  |  |
|  | 3/5 | 1 | 0.263157895 | 3/4 | 0 | -0.125 | 3/4 | -0.2 | -0.090909091 |  |  |  |
|  | 3/6 | 1.5 | 0.375 | 3/5 | 0.333333333 | 0.142857143 | 3/5 | 0 | -0.2 |  |  |  |
|  | 3/7 | 0 | 0.121212121 | 3/6 | -0.25 | 0 | 3/6 | 0 | 0.25 |  |  |  |
|  | 3/8 | 0.6 | 0.027027027 | 3/7 | -0.333333333 | -0.5 | 3/7 | 0.5 | -0.1 |  |  |  |
|  | 3/9 | 0.125 | 0.184210526 | 3/8 | -0.5 | 0 | 3/8 | 0 | 0 |  |  |  |
|  | 3/10 | 0.444444444 | 0.022222222 | 3/9 | 0 | -0.25 | 3/9 | 0 | 0 |  |  |  |
|  | 3/11 | 0.384615385 | 0.02173913 | 3/10 | 1 | 0 | 3/10 | 0.5 | 0.222222222 |  |  |  |
|  | **Date** | **Singapore Imported** | **Singapore Local** | **Date** | **Taiwan Imported** | **Taiwan Local** | **Date** | **Hong Kong Imported** | **Hong Kong Local** | **Date** | **South Korea Imported** | **South Korea Local** |
| 3rd Wave | 3/4 |  | -0.05 | 2/29 | 1 | 0.571428571 | 3/1 | 0.125 | -0.058823529 |  |  |  |
|  | 3/5 | 1 | 0.263157895 | 3/1 | 0.5 | -0.181818182 | 3/2 | -0.444444444 | -0.0625 |  |  |  |
|  | 3/6 | 1.5 | 0.375 | 3/2 | 0 | -0.111111111 | 3/3 | 0 | -0.266666667 |  |  |  |
|  | 3/7 | 0 | 0.121212121 | 3/3 | 0 | 0 | 3/4 | -0.2 | -0.090909091 |  |  |  |
|  | 3/8 | 0.6 | 0.027027027 | 3/4 | 0 | -0.125 | 3/5 | 0 | -0.2 |  |  |  |
|  | 3/9 | 0.125 | 0.184210526 | 3/5 | 0.333333333 | 0.142857143 | 3/6 | 0 | 0.25 |  |  |  |
|  | 3/10 | 0.444444444 | 0.022222222 | 3/6 | -0.25 | 0 | 3/7 | 0.5 | -0.1 |  |  |  |
|  | 3/11 | 0.384615385 | 0.02173913 | 3/7 | -0.333333333 | -0.5 | 3/8 | 0 | 0 |  |  |  |
|  | 3/12 | 0.333333333 | -0.106382979 | 3/8 | -0.5 | 0 | 3/9 | 0 | 0 |  |  |  |
|  | 3/13 | 0.291666667 | -0.166666667 | 3/9 | 0 | -0.25 | 3/10 | 0.5 | 0.222222222 |  |  |  |
|  | 3/14 | 0.322580645 | -0.057142857 | 3/10 | 1 | 0 | 3/11 | 0.777777778 | -0.181818182 |  |  |  |
|  | 3/15 | 0.170731707 | -0.060606061 | 3/11 | 0.5 | 0 | 3/12 | 0.125 | 0 |  |  |  |
|  | 3/16 | 0.104166667 | -0.129032258 | 3/12 | 0 | -0.333333333 | 3/13 | 0.111111111 | 0.111111111 |  |  |  |
|  | 3/17 | 0.566037736 | 0.259259259 | 3/13 | 0 | 0 | 3/14 | 0.05 | 0.1 |  |  |  |
|  | **Date** | **Singapore Imported** | **Singapore Local** | **Date** | **Taiwan Imported** | **Taiwan Local** | **Date** | **Hong Kong Imported** | **Hong Kong Local** | **Date** | **South Korea Imported** | **South Korea Local** |
| 4th Wave | 3/17 | 0.566037736 | 0.259259259 | 3/15 | 1 | 0 | 3/15 | 0.19047619 | -0.181818182 | 3/23 |  |  |
|  | 3/18 | 0.21686747 | 0.294117647 | 3/16 | 0.666666667 | 0 | 3/16 | 0.28 | 0.111111111 | 3/24 | 0.234042553 | -0.047543582 |
|  | 3/19 | 0.207920792 | 0.181818182 | 3/17 | 0.45 | -0.5 | 3/17 | 0.09375 | 0.2 | 3/25 | 0.396551724 | -0.064891847 |
|  | 3/20 | 0.213114754 | 0.096153846 | 3/18 | 0.689655172 | 2 | 3/18 | 0.142857143 | 0.916666667 | 3/26 | 0.265432099 | -0.161921708 |
|  | 3/21 | 0.168918919 | 0.035087719 | 3/19 | 0.12244898 | 0.333333333 | 3/19 | 0.2 | 0.260869565 | 3/27 | 0.087804878 | -0.029723992 |
|  | 3/22 | 0.208092486 | 0.050847458 | 3/20 | 0.436363636 | 0.5 | 3/20 | 0.458333333 | 0.689655172 | 3/28 | 0.179372197 | -0.089715536 |
|  | 3/23 | 0.057416268 | 0.064516129 | 3/21 | 0.189873418 | 0 | 3/21 | 0.085714286 | 0.142857143 | 3/29 | 0.098859316 | -0.045673077 |
|  | 3/24 | 0.036199095 | 0.318181818 | 3/22 | 0.074468085 | 0.5 | 3/22 | 0.171052632 | 0.428571429 | 3/30 | 0.148788927 | -0.073047859 |
|  | 3/25 | 0.03930131 | 0.126436782 | 3/23 | 0.168316832 | 0.111111111 | 3/23 | 0.157303371 | 0.2 | 3/31 | 0.045180723 | 0.092391304 |
|  | 3/26 | -0.012605042 | 0.183673469 | 3/24 | 0.084745763 | 0.1 | 3/24 | 0.067961165 | 0.135416667 | 4/1 | -0.040345821 | 0.037313433 |
|  | 3/27 | 0.025531915 | 0.146551724 | 3/25 | -0.0078125 | -0.181818182 | 3/25 | 0.009090909 | -0.018348624 | 4/2 | -0.048048048 | 0.002398082 |
|  | 3/28 | -0.058091286 | 0.157894737 | 3/26 | 0.05511811 | 0.111111111 | 3/26 | 0.135135135 | 0.112149533 | 4/3 | 0.066246057 | -0.062200957 |
|  | 3/29 | -0.136563877 | 0.090909091 | 3/27 | -0.067164179 | -0.3 | 3/27 | 0.079365079 | 0.058823529 | 4/4 | -0.038461538 | -0.099489796 |
|  | 3/30 | -0.010204082 | 0.095238095 | 3/28 | -0.032 | 0.285714286 | 3/28 | 0.257352941 | 0.095238095 | 4/5 | 0.012307692 | -0.079320113 |
|  | **Date** | **Singapore Imported** | **Singapore Local** | **Date** | **Taiwan Imported** | **Taiwan Local** | **Date** | **Hong Kong Imported** | **Hong Kong Local** | **Date** | **South Korea Imported** | **South Korea Local** |
| 5th Wave | 3/22 | 0.208092486 | 0.050847458 | 3/19 | 0.12244898 | 0.333333333 | 3/20 | 0.458333333 | 0.689655172 | 4/2 | -0.048048048 | 0.002398082 |
|  | 3/23 | 0.057416268 | 0.064516129 | 3/20 | 0.436363636 | 0.5 | 3/21 | 0.085714286 | 0.142857143 | 4/3 | 0.066246057 | -0.062200957 |
|  | 3/24 | 0.036199095 | 0.318181818 | 3/21 | 0.189873418 | 0 | 3/22 | 0.171052632 | 0.428571429 | 4/4 | -0.038461538 | -0.099489796 |
|  | 3/25 | 0.03930131 | 0.126436782 | 3/22 | 0.074468085 | 0.5 | 3/23 | 0.157303371 | 0.2 | 4/5 | 0.012307692 | -0.079320113 |
|  | 3/26 | -0.012605042 | 0.183673469 | 3/23 | 0.168316832 | 0.111111111 | 3/24 | 0.067961165 | 0.135416667 | 4/6 |  |  |
|  | 3/27 | 0.025531915 | 0.146551724 | 3/24 | 0.084745763 | 0.1 | 3/25 | 0.009090909 | -0.018348624 | 4/7 |  |  |
|  | 3/28 | -0.058091286 | 0.157894737 | 3/25 | -0.0078125 | -0.181818182 | 3/26 | 0.135135135 | 0.112149533 | 4/8 |  |  |
|  | 3/29 | -0.136563877 | 0.090909091 | 3/26 | 0.05511811 | 0.111111111 | 3/27 | 0.079365079 | 0.058823529 | 4/9 |  |  |
|  | 3/30 | -0.010204082 | 0.095238095 | 3/27 | -0.067164179 | -0.3 | 3/28 | 0.257352941 | 0.095238095 | 4/10 |  |  |
|  | 3/31 | -0.12371134 | 0.048913043 | 3/28 | -0.032 | 0.285714286 | 3/29 | 0.099415205 | -0.014492754 | 4/11 |  |  |
|  | 4/1 | -0.135294118 | 0.134715026 | 3/29 | 0.008264463 | -0.222222222 | 3/30 | 0.058510638 | -0.066176471 | 4/12 |  |  |
|  | 4/2 | -0.115646259 | 0.082191781 | 3/30 | -0.147540984 | -2.0462E-16 | 3/31 | 0.050251256 | -0.062992126 | 4/13 |  |  |
|  | 4/3 | -0.253846154 | 0.210970464 | 3/31 | -0.057692308 | 0.142857143 | 4/1 | 0.071770335 | 0.100840336 | 4/14 |  |  |
|  | 4/4 | -0.195876289 | -0.024390244 | 4/1 | -0.132653061 | 0 | 4/2 | 0.013392857 | -0.06870229 | 4/15 |  |  |

Table S11: Rate of change in cases 7 days post intervention (Calculated using 14-day moving average for daily cases)

| **7 Days Post Intervention** | | | | | | | | | | | | |
| --- | --- | --- | --- | --- | --- | --- | --- | --- | --- | --- | --- | --- |
|  | **Date** | **Singapore Imported** | **Singapore Local** | **Date** | **Taiwan Imported** | **Taiwan Local** | **Date** | **Hong Kong Imported** | **Hong Kong Local** | **Date** | **South Korea Imported** | **South Korea Local** |
| 1st Wave | 1/24 |  |  | 1/26 |  |  | 1/28 |  |  | 2/5 | 0.25 | 0.428571429 |
|  | 1/25 |  |  | 1/27 |  |  | 1/29 |  |  | 2/6 | 0.1 | 0.2 |
|  | 1/26 |  |  | 1/28 |  |  | 1/30 |  |  | 2/7 | -0.090909091 | 0 |
|  | 1/27 |  |  | 1/29 |  |  | 1/31 |  |  | 2/8 | 0 | 0 |
|  | 1/28 |  |  | 1/30 |  |  | 2/1 |  |  | 2/9 | 0.1 | 0.083333333 |
|  | 1/29 |  |  | 1/31 |  |  | 2/2 |  |  | 2/10 | -0.090909091 | 0.076923077 |
|  | 1/30 |  |  | 2/1 |  |  | 2/3 |  |  | 2/11 | 0 | 0 |
|  | **Date** | **Singapore Imported** | **Singapore Local** | **Date** | **Taiwan Imported** | **Taiwan Local** | **Date** | **Hong Kong Imported** | **Hong Kong Local** | **Date** | **South Korea Imported** | **South Korea Local** |
| 2nd Wave | 2/27 | 0 | -0.153846154 | 2/26 |  | 0.076923077 | 2/26 | 0.4 | 0.096774194 | NA |  |  |
|  | 2/28 | 0 | -0.090909091 | 2/27 |  | 0 | 2/27 | 0 | -0.029411765 | NA |  |  |
|  | 2/29 | 0 | -0.133333333 | 2/28 |  | 0.071428571 | 2/28 | 0.142857143 | -0.090909091 | NA |  |  |
|  | 3/1 | -0.5 | 0.153846154 | 2/29 | 1 | 0.133333333 | 2/29 | 0 | 0.033333333 | NA |  |  |
|  | 3/2 | 0 | -0.033333333 | 3/1 | 0.5 | 0 | 3/1 | 0.25 | 0.064516129 | NA |  |  |
|  | 3/3 | 0 | -0.034482759 | 3/2 | 0 | -0.058823529 | 3/2 | 0 | -0.060606061 | NA |  |  |
|  | 3/4 | 1 | 0 | 3/3 | 0 | 0.0625 | 3/3 | 0 | -0.064516129 | NA |  |  |
|  | **Date** | **Singapore Imported** | **Singapore Local** | **Date** | **Taiwan Imported** | **Taiwan Local** | **Date** | **Hong Kong Imported** | **Hong Kong Local** | **Date** | **South Korea Imported** | **South Korea Local** |
| 3rd Wave | 3/4 | 1 | 0 | 2/29 | 1 | 0.133333333 | 3/1 | 0.25 | 0.064516129 | NA |  |  |
|  | 3/5 | 0.5 | 0.214285714 | 3/1 | 0.5 | 0 | 3/2 | 0 | -0.060606061 | NA |  |  |
|  | 3/6 | 0.666666667 | 0.323529412 | 3/2 | 0 | -0.058823529 | 3/3 | 0 | -0.064516129 | NA |  |  |
|  | 3/7 | 0 | 0.088888889 | 3/3 | 0 | 0.0625 | 3/4 | 0.1 | 0 | NA |  |  |
|  | 3/8 | 0.6 | 0.102040816 | 3/4 | 0 | -0.117647059 | 3/5 | 0 | -0.137931034 | NA |  |  |
|  | 3/9 | 0.125 | 0.166666667 | 3/5 | 0.333333333 | 0.066666667 | 3/6 | 0.090909091 | 0.08 | NA |  |  |
|  | 3/10 | 0.444444444 | 0.047619048 | 3/6 | 0 | -0.0625 | 3/7 | 0.166666667 | -0.037037037 | NA |  |  |
|  | **Date** | **Singapore Imported** | **Singapore Local** | **Date** | **Taiwan Imported** | **Taiwan Local** | **Date** | **Hong Kong Imported** | **Hong Kong Local** | **Date** | **South Korea Imported** | **South Korea Local** |
| 4th Wave | 3/17 | 0.548387097 | 0.111111111 | 3/15 | 0.625 | 0 | 3/15 | 0.148148148 | -0.1 | 3/23 |  |  |
|  | 3/18 | 0.239583333 | 0.1375 | 3/16 | 0.615384615 | -0.166666667 | 3/16 | 0.225806452 | 0.055555556 | 3/24 |  |  |
|  | 3/19 | 0.226890756 | 0.032967033 | 3/17 | 0.476190476 | -0.2 | 3/17 | 0.157894737 | 0.210526316 | 3/25 |  |  |
|  | 3/20 | 0.226027397 | -0.021276596 | 3/18 | 0.677419355 | 0.5 | 3/18 | 0.272727273 | 0.391304348 | 3/26 |  |  |
|  | 3/21 | 0.195530726 | 0 | 3/19 | 0.115384615 | -1.19361E-16 | 3/19 | 0.178571429 | 0.1875 | 3/27 |  |  |
|  | 3/22 | 0.200934579 | 0.010869565 | 3/20 | 0.413793103 | 0.333333333 | 3/20 | 0.363636364 | 0.552631579 | 3/28 |  |  |
|  | 3/23 | 0.06614786 | 0 | 3/21 | 0.219512195 | 0 | 3/21 | 0.077777778 | 0.13559322 | 3/29 |  |  |
|  | **Date** | **Singapore Imported** | **Singapore Local** | **Date** | **Taiwan Imported** | **Taiwan Local** | **Date** | **Hong Kong Imported** | **Hong Kong Local** | **Date** | **South Korea Imported** | **South Korea Local** |
| 5th Wave | 3/22 | 0.200934579 | 0.010869565 | 3/19 | 0.115384615 | -1.19361E-16 | 3/20 | 0.363636364 | 0.552631579 | 4/2 | 0.054545455 | -0.091930541 |
|  | 3/23 | 0.06614786 | 0 | 3/20 | 0.413793103 | 0.333333333 | 3/21 | 0.077777778 | 0.13559322 | 4/3 | 0.074712644 | -0.044994376 |
|  | 3/24 | 0.138686131 | 0.301075269 | 3/21 | 0.219512195 | 0 | 3/22 | 0.175257732 | 0.328358209 | 4/4 | 0.048128342 | -0.094228504 |
|  | 3/25 | 0.086538462 | 0.173553719 | 3/22 | 0.13 | 0.375 | 3/23 | 0.184210526 | 0.191011236 | 4/5 | 0.051020408 | -0.061118336 |
|  | 3/26 | 0.053097345 | 0.183098592 | 3/23 | 0.221238938 | 0.090909091 | 3/24 | 0.074074074 | 0.141509434 | 4/6 |  |  |
|  | 3/27 | 0.089635854 | 0.130952381 | 3/24 | 0.137681159 | 0 | 3/25 | 0.04137931 | 0.074380165 | 4/7 |  |  |
|  | 3/28 | 0.028277635 | 0.121052632 | 3/25 | 0.121019108 | 0 | 3/26 | 0.152317881 | 0.138461538 | 4/8 |  |  |

Table S12: Rate of change in cases 14 days post intervention (Calculated using 14-day moving average for daily cases)

| **14 Days Post Intervention** | | | | | | | | | | | | |
| --- | --- | --- | --- | --- | --- | --- | --- | --- | --- | --- | --- | --- |
|  | **Date** | **Singapore Imported** | **Singapore Local** | **Date** | **Taiwan Imported** | **Taiwan Local** | **Date** | **Hong Kong Imported** | **Hong Kong Local** | **Date** | **South Korea Imported** | **South Korea Local** |
| 1st Wave | 1/24 |  |  | 1/26 |  |  | 1/28 |  |  | 2/5 | 0.25 | 0.428571429 |
|  | 1/25 |  |  | 1/27 |  |  | 1/29 |  |  | 2/6 | 0.1 | 0.2 |
|  | 1/26 |  |  | 1/28 |  |  | 1/30 |  |  | 2/7 | -0.090909091 | 0 |
|  | 1/27 |  |  | 1/29 |  |  | 1/31 |  |  | 2/8 | 0 | 0 |
|  | 1/28 |  |  | 1/30 |  |  | 2/1 |  |  | 2/9 | 0.1 | 0.083333333 |
|  | 1/29 |  |  | 1/31 |  |  | 2/2 |  |  | 2/10 | -0.090909091 | 0.076923077 |
|  | 1/30 |  |  | 2/1 |  |  | 2/3 |  |  | 2/11 | 0 | 0 |
|  | 1/31 |  |  | 2/2 |  |  | 2/4 |  |  | 2/12 | 0 | 0 |
|  | 2/1 |  |  | 2/3 |  |  | 2/5 |  |  | 2/13 | -0.2 | -0.071428571 |
|  | 2/2 |  |  | 2/4 | 0 | 0 | 2/6 | -0.181818182 | 0.3 | 2/14 | -0.125 | -0.230769231 |
|  | 2/3 |  |  | 2/5 | 0 | 0 | 2/7 | -0.111111111 | 0 | 2/15 | -0.142857143 | 0 |
|  | 2/4 |  |  | 2/6 | 0.625 | 0 | 2/8 | 0 | 0 | 2/16 | 0 | -0.1 |
|  | 2/5 |  |  | 2/7 | -0.153846154 | 0 | 2/9 | -0.375 | 0.769230769 | 2/17 | 0 | 0 |
|  | 2/6 | -0.047619048 | 0.5 | 2/8 | 0.090909091 | 0 | 2/10 | 0 | 0.260869565 | 2/18 | 0 | 0 |
|  | **Date** | **Singapore Imported** | **Singapore Local** | **Date** | **Taiwan Imported** | **Taiwan Local** | **Date** | **Hong Kong Imported** | **Hong Kong Local** | **Date** | **South Korea Imported** | **South Korea Local** |
| 2nd Wave | 2/27 | 0 | -0.153846154 | 2/26 |  | 0.076923077 | 2/26 | 0.4 | 0.096774194 |  |  |  |
|  | 2/28 | 0 | -0.090909091 | 2/27 |  | 0 | 2/27 | 0 | -0.029411765 |  |  |  |
|  | 2/29 | 0 | -0.133333333 | 2/28 |  | 0.071428571 | 2/28 | 0.142857143 | -0.090909091 |  |  |  |
|  | 3/1 | -0.5 | 0.153846154 | 2/29 | 1 | 0.133333333 | 2/29 | 0 | 0.033333333 |  |  |  |
|  | 3/2 | 0 | -0.033333333 | 3/1 | 0.5 | 0 | 3/1 | 0.25 | 0.064516129 |  |  |  |
|  | 3/3 | 0 | -0.034482759 | 3/2 | 0 | -0.058823529 | 3/2 | 0 | -0.060606061 |  |  |  |
|  | 3/4 | 1 | 0 | 3/3 | 0 | 0.0625 | 3/3 | 0 | -0.064516129 |  |  |  |
|  | 3/5 | 0.5 | 0.214285714 | 3/4 | 0 | -0.117647059 | 3/4 | 0.1 | 0 |  |  |  |
|  | 3/6 | 0.666666667 | 0.323529412 | 3/5 | 0.333333333 | 0.066666667 | 3/5 | 0 | -0.137931034 |  |  |  |
|  | 3/7 | 0 | 0.088888889 | 3/6 | 0 | -0.0625 | 3/6 | 0.090909091 | 0.08 |  |  |  |
|  | 3/8 | 0.6 | 0.102040816 | 3/7 | 0 | 0 | 3/7 | 0.166666667 | -0.037037037 |  |  |  |
|  | 3/9 | 0.125 | 0.166666667 | 3/8 | 0 | -0.133333333 | 3/8 | 0.071428571 | -0.038461538 |  |  |  |
|  | 3/10 | 0.444444444 | 0.047619048 | 3/9 | 0 | -0.153846154 | 3/9 | -0.266666667 | -0.04 |  |  |  |
|  | 3/11 | 0.461538462 | 0 | 3/10 | 0.25 | 0 | 3/10 | 0.272727273 | -0.083333333 |  |  |  |
|  | **Date** | **Singapore Imported** | **Singapore Local** | **Date** | **Taiwan Imported** | **Taiwan Local** | **Date** | **Hong Kong Imported** | **Hong Kong Local** | **Date** | **South Korea Imported** | **South Korea Local** |
| 3rd Wave | 3/4 | 1 | 0 | 2/29 | 1 | 0.133333333 | 3/1 | 0.25 | 0.064516129 |  |  |  |
|  | 3/5 | 0.5 | 0.214285714 | 3/1 | 0.5 | 0 | 3/2 | 0 | -0.060606061 |  |  |  |
|  | 3/6 | 0.666666667 | 0.323529412 | 3/2 | 0 | -0.058823529 | 3/3 | 0 | -0.064516129 |  |  |  |
|  | 3/7 | 0 | 0.088888889 | 3/3 | 0 | 0.0625 | 3/4 | 0.1 | 0 |  |  |  |
|  | 3/8 | 0.6 | 0.102040816 | 3/4 | 0 | -0.117647059 | 3/5 | 0 | -0.137931034 |  |  |  |
|  | 3/9 | 0.125 | 0.166666667 | 3/5 | 0.333333333 | 0.066666667 | 3/6 | 0.090909091 | 0.08 |  |  |  |
|  | 3/10 | 0.444444444 | 0.047619048 | 3/6 | 0 | -0.0625 | 3/7 | 0.166666667 | -0.037037037 |  |  |  |
|  | 3/11 | 0.461538462 | 0 | 3/7 | 0 | 0 | 3/8 | 0.071428571 | -0.038461538 |  |  |  |
|  | 3/12 | 0.368421053 | 0 | 3/8 | 0 | -0.133333333 | 3/9 | -0.266666667 | -0.04 |  |  |  |
|  | 3/13 | 0.384615385 | 0.03030303 | 3/9 | 0 | -0.153846154 | 3/10 | 0.272727273 | -0.083333333 |  |  |  |
|  | 3/14 | 0.277777778 | 0.029411765 | 3/10 | 0.25 | 0 | 3/11 | 0.428571429 | -0.136363636 |  |  |  |
|  | 3/15 | 0.217391304 | -0.014285714 | 3/11 | 0.2 | -0.090909091 | 3/12 | 0.1 | -0.105263158 |  |  |  |
|  | 3/16 | 0.107142857 | 0.043478261 | 3/12 | 0.166666667 | 0 | 3/13 | 0.090909091 | 0.176470588 |  |  |  |
|  | 3/17 | 0.548387097 | 0.111111111 | 3/13 | -0.142857143 | 0 | 3/14 | 0.125 | 0 |  |  |  |
|  | **Date** | **Singapore Imported** | **Singapore Local** | **Date** | **Taiwan Imported** | **Taiwan Local** | **Date** | **Hong Kong Imported** | **Hong Kong Local** | **Date** | **South Korea Imported** | **South Korea Local** |
| 4th Wave | 3/17 | 0.548387097 | 0.111111111 | 3/15 | 0.625 | 0 | 3/15 | 0.148148148 | -0.1 | 3/23 |  |  |
|  | 3/18 | 0.239583333 | 0.1375 | 3/16 | 0.615384615 | -0.166666667 | 3/16 | 0.225806452 | 0.055555556 | 3/24 |  |  |
|  | 3/19 | 0.226890756 | 0.032967033 | 3/17 | 0.476190476 | -0.2 | 3/17 | 0.157894737 | 0.210526316 | 3/25 |  |  |
|  | 3/20 | 0.226027397 | -0.021276596 | 3/18 | 0.677419355 | 0.5 | 3/18 | 0.272727273 | 0.391304348 | 3/26 |  |  |
|  | 3/21 | 0.195530726 | 0 | 3/19 | 0.115384615 | -1.19361E-16 | 3/19 | 0.178571429 | 0.1875 | 3/27 |  |  |
|  | 3/22 | 0.200934579 | 0.010869565 | 3/20 | 0.413793103 | 0.333333333 | 3/20 | 0.363636364 | 0.552631579 | 3/28 |  |  |
|  | 3/23 | 0.06614786 | 0 | 3/21 | 0.219512195 | 0 | 3/21 | 0.077777778 | 0.13559322 | 3/29 |  |  |
|  | 3/24 | 0.138686131 | 0.301075269 | 3/22 | 0.13 | 0.375 | 3/22 | 0.175257732 | 0.328358209 | 3/30 |  |  |
|  | 3/25 | 0.086538462 | 0.173553719 | 3/23 | 0.221238938 | 0.090909091 | 3/23 | 0.184210526 | 0.191011236 | 3/31 | 0.08685446 | 0.004004004 |
|  | 3/26 | 0.053097345 | 0.183098592 | 3/24 | 0.137681159 | 0 | 3/24 | 0.074074074 | 0.141509434 | 4/1 | 0.069114471 | -0.023928215 |
|  | 3/27 | 0.089635854 | 0.130952381 | 3/25 | 0.121019108 | 0 | 3/25 | 0.04137931 | 0.074380165 | 4/2 | 0.054545455 | -0.091930541 |
|  | 3/28 | 0.028277635 | 0.121052632 | 3/26 | 0.073863636 | 0.166666667 | 3/26 | 0.152317881 | 0.138461538 | 4/3 | 0.074712644 | -0.044994376 |
|  | 3/29 | 0.0125 | 0.079812207 | 3/27 | 0.079365079 | -0.071428571 | 3/27 | 0.183908046 | 0.182432432 | 4/4 | 0.048128342 | -0.094228504 |
|  | 3/30 | 0.024691358 | 0.086956522 | 3/28 | 0.053921569 | 0.153846154 | 3/28 | 0.199029126 | 0.108571429 | 4/5 | 0.051020408 | -0.061118336 |
|  | **Date** | **Singapore Imported** | **Singapore Local** | **Date** | **Taiwan Imported** | **Taiwan Local** | **Date** | **Hong Kong Imported** | **Hong Kong Local** | **Date** | **South Korea Imported** | **South Korea Local** |
| 5th Wave | 3/22 | 0.200934579 | 0.010869565 | 3/19 | 0.115384615 | -1.19361E-16 | 3/20 | 0.363636364 | 0.552631579 | 4/2 | 0.054545455 | -0.091930541 |
|  | 3/23 | 0.06614786 | 0 | 3/20 | 0.413793103 | 0.333333333 | 3/21 | 0.077777778 | 0.13559322 | 4/3 | 0.074712644 | -0.044994376 |
|  | 3/24 | 0.138686131 | 0.301075269 | 3/21 | 0.219512195 | 0 | 3/22 | 0.175257732 | 0.328358209 | 4/4 | 0.048128342 | -0.094228504 |
|  | 3/25 | 0.086538462 | 0.173553719 | 3/22 | 0.13 | 0.375 | 3/23 | 0.184210526 | 0.191011236 | 4/5 | 0.051020408 | -0.061118336 |
|  | 3/26 | 0.053097345 | 0.183098592 | 3/23 | 0.221238938 | 0.090909091 | 3/24 | 0.074074074 | 0.141509434 | 4/6 |  |  |
|  | 3/27 | 0.089635854 | 0.130952381 | 3/24 | 0.137681159 | 0 | 3/25 | 0.04137931 | 0.074380165 | 4/7 |  |  |
|  | 3/28 | 0.028277635 | 0.121052632 | 3/25 | 0.121019108 | 0 | 3/26 | 0.152317881 | 0.138461538 | 4/8 |  |  |
|  | 3/29 | 0.0125 | 0.079812207 | 3/26 | 0.073863636 | 0.166666667 | 3/27 | 0.183908046 | 0.182432432 | 4/9 |  |  |
|  | 3/30 | 0.024691358 | 0.086956522 | 3/27 | 0.079365079 | -0.071428571 | 3/28 | 0.199029126 | 0.108571429 | 4/10 |  |  |
|  | 3/31 | -0.038554217 | 0.12 | 3/28 | 0.053921569 | 0.153846154 | 3/29 | 0.12145749 | 0.113402062 | 4/11 |  |  |
|  | 4/1 | -0.035087719 | 0.132142857 | 3/29 | 0.037209302 | 0.066666667 | 3/30 | 0.090252708 | 0.032407407 | 4/12 |  |  |
|  | 4/2 | -0.051948052 | 0.113564669 | 3/30 | -0.004484305 | 0.0625 | 3/31 | 0.056291391 | 0.022421525 | 4/13 |  |  |
|  | 4/3 | -0.073972603 | 0.1898017 | 3/31 | 0.018018018 | 0.117647059 | 4/1 | 0.05015674 | 0.043859649 | 4/14 |  |  |
|  | 4/4 | -0.097633136 | 0.033333333 | 4/1 | -0.061946903 | -0.105263158 | 4/2 | 0.053731343 | 0.012605042 | 4/15 |  |  |
